# Hybrid gene misregulation in multiple developing tissues within a recent adaptive radiation of *Cyprinodon* pupfishes

**DOI:** 10.1371/journal.pone.0218899

**Published:** 2019-07-10

**Authors:** Joseph A. McGirr, Christopher H. Martin

**Affiliations:** 1 Department of Biology, University of North Carolina at Chapel Hill, Chapel Hill, North Carolina, United States of America; 2 Department of Integrative Biology and Museum of Vertebrate Zoology, University of California, Berkeley, California, United States of America; University of Arkansas, UNITED STATES

## Abstract

Genetic incompatibilities constitute the final stages of reproductive isolation and speciation, but little is known about incompatibilities that occur within recent adaptive radiations among closely related diverging populations. Crossing divergent species to form hybrids can break up coadapted variation, resulting in genetic incompatibilities within developmental networks shaping divergent adaptive traits. We crossed two closely related sympatric *Cyprinodon* pupfish species–a dietary generalist and a specialized molluscivore–and measured expression levels in their F1 hybrids to identify regulatory variation underlying the novel craniofacial morphology found in this recent microendemic adaptive radiation. We extracted mRNA from eight day old whole-larvae tissue and from craniofacial tissues dissected from 17–20 day old larvae to compare gene expression between a total of seven F1 hybrids and 24 individuals from parental species populations. We found 3.9% of genes differentially expressed between generalists and molluscivores in whole-larvae tissues and 0.6% of genes differentially expressed in craniofacial tissue. We found that 2.1% of genes were misregulated in whole-larvae hybrids whereas 19.1% of genes were misregulated in hybrid craniofacial tissues, after correcting for sequencing biases. We also measured allele specific expression across 15,429 heterozygous sites to identify putative compensatory regulatory mechanisms underlying differential expression between generalists and molluscivores. Together, our results highlight the importance of considering misregulation as an early indicator of genetic incompatibilities in the context of rapidly diverging adaptive radiations and suggests that compensatory regulatory divergence drives hybrid gene misregulation in developing tissues that give rise to novel craniofacial traits.

## Introduction

Changes in gene expression are an important source of variation in adaptive morphological traits [1–3]. As genetic variation accumulates in regulatory and coding sequences, stabilizing selection on gene expression results in coevolution such that molecular functions are largely maintained [4,5]. Crossing divergent species to form F1 hybrids can break up such coadapted variation, resulting in genetic incompatibilities within developing tissues that give rise to adaptive traits [6–8]. Genetic incompatibilities that reduce hybrid fitness can drive reproductive isolation either intrinsically–causing sterility or increased embryonic mortality–or extrinsically whereby incompatibilities reduce hybrid performance in a particular environment [9,10].

Of particular importance to the process of speciation are genetic incompatibilities caused by hybrid misregulation–transgressive expression levels in hybrids that are higher or lower than both parental species [6,11–15]. This pattern of expression causes Dobzhansky-Muller incompatibilities (DMIs) if incompatible alleles in hybrids cause misregulation that results in reduced hybrid fitness and thus increased postzygotic reproductive isolation [10,12,14,16–19]. Laboratory studies searching for genes that cause DMIs often identify genes causing sterility or embryonic lethality in hybrids. This approach ignores the fitness consequences of misregulation occurring at later developmental stages within diverse tissue types, thus underestimating the actual number of genetic incompatibilities distinguishing species [20,21]. Combining findings from these studies with analyses of hybrid misregulation in tissues that give rise to adaptive morphological traits can reveal a broader view of incompatibilities that arise during speciation.

Studies of gene expression in hybrids can also implicate regulatory mechanisms underlying expression divergence between parental species, which is important for understanding how expression levels are inherited and how they shape adaptive traits [22–24]. Research on hybrid gene expression thus far has shown mixed results regarding patterns of inheritance [25]. Some studies found evidence for ubiquitous transgressive expression inherited in F1 hybrids (i.e. over- or under-dominance) [11,13,26], while others found predominately additive patterns [19,27,28]. Mechanisms of gene expression divergence in F1 hybrids are characterized as interactions between *cis*-regulatory elements and *trans*-regulatory factors. *Cis* elements are often non-coding regions of DNA proximal to genes that are bound by *trans*-acting proteins and RNAs to regulate mRNA abundance. It is possible to identify mechanisms of gene expression divergence between parental species by bringing *cis* elements from both parents together in the same *trans* environment in F1 hybrids and quantifying allele specific expression (ASE) of parental alleles at heterozygous sites [22,29]. *Cis* and *trans* regulatory variants can compensate for one another if stabilizing selection favors an optimal level of gene expression. Hybrid misregulation is expected when different compensatory variants have accumulated in diverging lineages [30–33].

**Fig 1:**
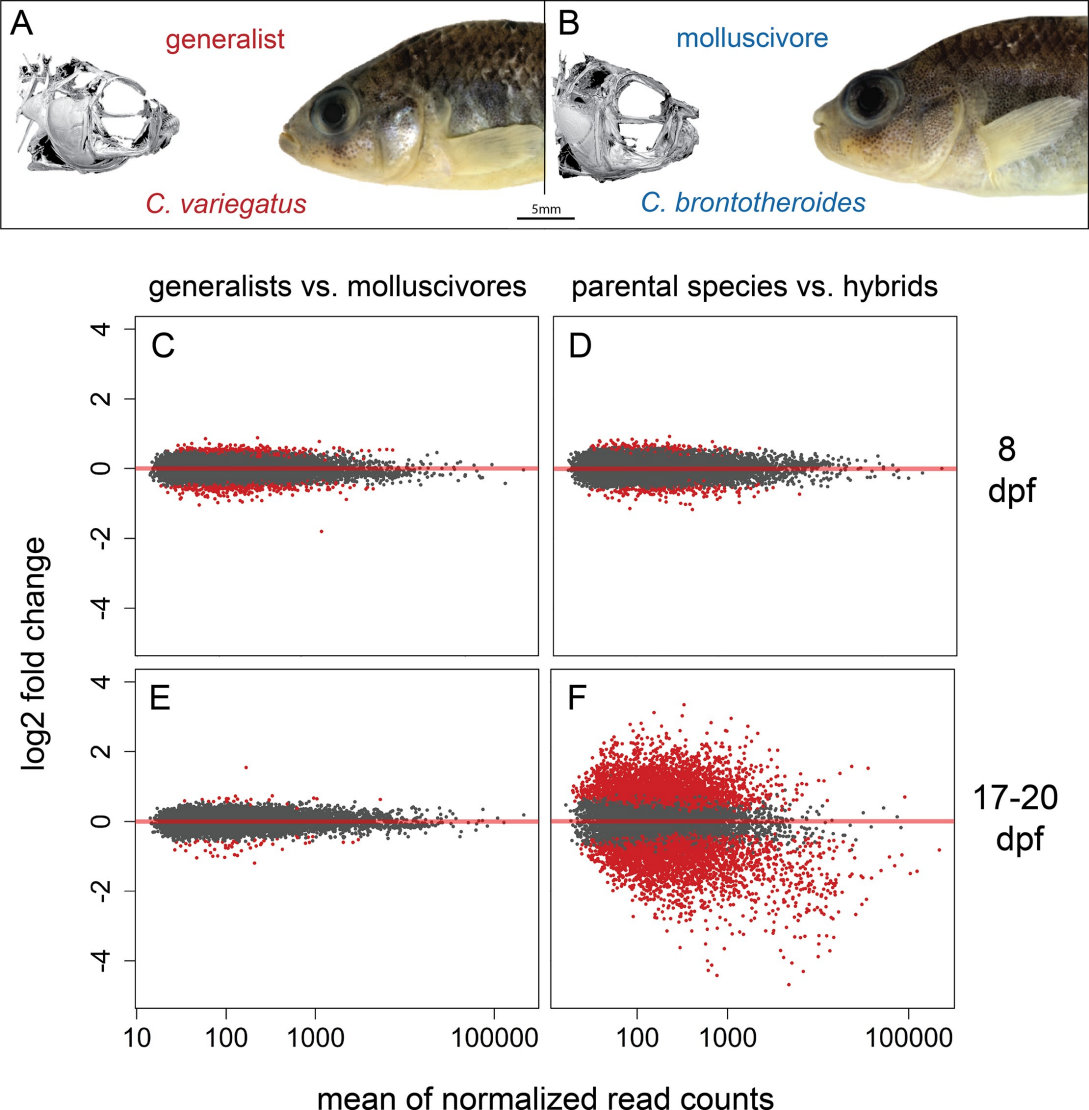
Extensive misregulation in F1 hybrid craniofacial tissues. (A) *Cyprinodon variegatus*–the generalist. (B) *C*. *brontotheroides–*the molluscivore (μCT scans of the cranial skeleton of each species modified from [66]. Variation in gene expression (C) between generalists vs. molluscivores 8 days post fertilization (dpf), (D) parental species vs. hybrids at 8 dpf, (E) generalists vs. molluscivores at 17–20 dpf, and (F) parental species vs. hybrids at 17–20 dpf. Red points indicate genes detected as differentially expressed at 5% false discovery rate with Benjamini-Hochberg multiple testing adjustment. Grey points indicate genes showing no significant difference in expression between groups. Red line indicates a log2 fold change of zero between groups. Points above/below the line are upregulated/downregulated in molluscivores relative to generalists (C and E) or hybrids relative to parental species (D and F).

Here we investigated F1 hybrids from crosses between two closely related species of *Cyprinodon* pupfishes to understand regulatory mechanisms that led to the evolution of novel craniofacial adaptations in this group (Fig 1A). *Cyprinodon brontotheroides*–hereafter referred to as the molluscivore–is a trophic specialist species endemic to San Salvador Island, Bahamas that has adapted to eat hard shelled prey including mollusks and ostracods [34,35]. This species likely diverged from a generalist common ancestor within the past 10,000 years to occupy this novel niche [36–40]. Adapting to this niche involved extreme morphological divergence in craniofacial traits compared to its sympatric generalist sister species *Cyprinodon variegatus* [35,41]. This species consumes mainly algae and detritus and is hereafter referred to as the ‘generalist.’ Almost all other Caribbean pupfish species are generalists, with the exception of a novel scale-eating pupfish that is also a member of the San Salvador pupfish radiation [35,38] and a second sympatric radiation of trophic specialists in Laguna Chichancanab, Mexico [42,43]. Molluscivores exhibit a novel skeletal protrusion on the anteriodorsal head of the maxilla not found in generalist populations that may be used to stabilize prey items held within its oral jaws, which are shorter and more robust relative to generalist species (Fig 1A). This jaw morphology provides higher mechanical advantage for crushing mollusks and other hard-shelled prey [38,44].

Molluscivores and generalists readily hybridize in the laboratory to produce fertile F1 offspring with approximately intermediate craniofacial morphologies and no obvious sex ratio distortion [38,45,46]. These species remain largely reproductively isolated in sympatry across multiple lake populations (genome-wide average Fst = 0.08; [45,47,48]). Therefore, unlike most studies of hybrid misregulation, we are not solely concerned with identifying gene expression patterns underlying hybrid sterility or lethality. Rather, we also aim to characterize misregulation in developing tissues that gives rise to novel craniofacial phenotypes within a young species pair with ongoing gene flow. We dissected craniofacial tissue from 17–20 day old F1 hybrids and extracted total mRNA to quantify gene expression levels. We also extracted whole-larvae mRNA from 8 day old generalists, molluscivores, and their F1 hybrids. We found genes misregulated in hybrids at both stages. Finally, we quantified allele specific expression (ASE) across exome-wide heterozygous sites to uncover mechanisms of regulatory divergence and found evidence for putative compensatory variation influencing patterns of hybrid misregulation.

## Materials and methods

### Ethics

Fishes were euthanized in an overdose of buffered MS-222 (Finquel, Inc.) following approved protocols from the University of California, Davis Institutional Animal Care and Use Committee (#17455) and University of California, Berkeley Animal Care and Use Committee (AUP-2015-01-7053). Field collections were permitted by the Bahamian Department of Agriculture (permit agr/nat/1).

### Study system and sample collection

Our methods for raising larvae and extracting RNA were identical to previously outlined methods [49]. We collected fishes for breeding from three hypersaline lakes on San Salvador Island, Bahamas (Little Lake, Osprey Lake, and Crescent Pond) using a hand net or seine net between 2011 and 2017. These fishes were reared at 25–27°C, 10–15 ppt salinity, pH 8.3, and fed a mix of commercial pellet foods and frozen foods. All lab bred larvae were raised exclusively on newly hatched brine shrimp after hatching and before euthanasia. Individuals were euthanized in an overdose of buffered MS‐222 and stored in RNA later (Ambion, Inc.) at -20°C for up to 18 months. We used RNeasy Mini Kits (Qiagen catalog #74104) to extract RNA from all samples.

We previously generated 24 transcriptomes belonging to generalists and molluscivores collected at two early developmental stages: 8–10 days post fertilization (dpf) and 17–20 dpf [49]. RNA was extracted from whole-larvae tissue at 8–10 dpf. We dissected all 17–20 dpf samples to extract RNA from anterior craniofacial tissues containing the dentary, angular, articular, maxilla, premaxilla, palatine, and associated craniofacial connective tissues (S1 Fig). Dissections were performed using fine‐tipped tweezers washed with RNase AWAY (Molecular BioProducts). These 24 samples were generated by breeding populations of lab-raised fishes that resulted from either one or two generations of full-sib breeding between wild caught fishes from Little Lake and Crescent Pond on San Salvador Island, Bahamas (Table 1). There was variation in sampling time because eggs were fertilized naturally within breeding tanks and collected on the same day or subsequent day following egg laying. We collected larvae in a haphazard manner over multiple spawning intervals and it is unlikely that sampling time varied consistently by species.

**Table 1:** Sampling design for mRNA sequencing. Parental fishes crossed to produce larvae for sequencing were either wild-caught (F0) or lab-raised over *n* generations (indicated by F*n*). Individuals were sampled eight days post fertilization (dpf), 8–10 dpf, or 17–20 dpf.

mothers	fathers	offspring sampled	lake population	sequencing round	stage (dpf)
F0 generalist	F0 molluscivore	3 hybrids	osprey lake	4	8
F0 generalists	F0 generalists	3 generalists	osprey lake	3	8
F0 molluscivores	F0 molluscivores	3 molluscivores	osprey lake	3	8
F0 generalists	F0 generalists	3 generalists	crescent pond	3	8
F0 molluscivores	F0 molluscivores	3 molluscivore	crescent pond	4	8
F1 generalists	F1 generalists	3 generalists	little lake	1	8–10
F2 molluscivores	F2 molluscivores	3 molluscivores	little lake	1	8–10
F2 generalists	F2 generalists	3 generalists	crescent pond	1	8–10
F2 molluscivores	F2 molluscivores	3 molluscivores	crescent pond	1	8–10
F2 generalist	F3 molluscivore	4 hybrids	little lake	2	17–20
F1 generalists	F1 generalists	3 generalists	little lake	1	17–20
F2 molluscivores	F2 molluscivores	3 molluscivores	little lake	1	17–20
F2 generalists	F2 generalists	3 generalists	crescent pond	1	17–20
F2 molluscivores	F2 molluscivores	3 molluscivores	crescent pond	1	17–20

Here we analyze an additional 19 transcriptomes from generalists, molluscivores, and their F1 hybrids (Table 1). First, we crossed a generalist female with a molluscivore male to generate four F1 hybrids that were collected at 17–20 dpf and extracted RNA from dissected craniofacial tissues. A lab-reared female generalist was used to generate hybrids that was derived from wild caught generalists from Little Lake following one generation of full-sib mating. A lab-reared male molluscivore was used to generate hybrids that was derived from wild caught molluscivores from Little Lake following two generations of full-sib mating.

We performed separate crosses to collect larvae at exactly 8 dpf (190–194 hours after fertilization rather than 8–10 days). We crossed a generalist female with a molluscivore male to generate three F1 hybrids for whole-larvae RNA extractions. The parents of these hybrids were wild-caught from Osprey Lake. Finally, we extracted whole-larvae RNA from six generalists and six molluscivores collected at 8 dpf. These samples were generated from wild-caught individuals from Osprey Lake and Crescent Pond. In total, we analyzed transcriptomes from 43 individuals that involved four separate rounds of sequencing (Table 1 and S1 Table).

### RNA sequencing and alignment

The previously reported 24 transcriptomes were sequenced at the High Throughput Genomic Sequencing Facility at UNC Chapel Hill in April 2017 [49]. The 24 libraries were prepared at the facility using the KAPA stranded mRNAseq kit (KAPA Biosystems 2016) followed by sequencing on one lane of Illumina 150 paired-end Hiseq4000 (Table 1 and S2 Table).

19 additional transcriptomes were sequenced at The Vincent J. Coates Genomics Sequencing Laboratory at the University of California, Berkeley. All 19 libraries were prepared at the facility using the Illumina stranded Truseq RNA kit (Illumina RS-122-2001) and all sequencing was performed on Illumina 150 paired-end Hiseq4000. Four libraries for RNA extracted from 17–20 dpf hybrid craniofacial tissues were pooled on a single lane and sequenced in June 2017. 15 libraries for whole-larvae RNA samples collected at exactly 8 dpf were pooled across one and three lanes and sequenced in May (n = 9) and July (n = 6) 2018, respectively (Table 1 and S1 Table).

We filtered all raw reads using Trim Galore (v. 4.4, Babraham Bioinformatics) to remove Illumina adaptors and low‐quality reads (mean Phred score < 20) and mapped filtered reads to the scaffolds of the *Cyprinodon* reference genome (NCBI, *C*. *variegatus* annotation release 100, total sequence length = 1,035,184,475; number of scaffolds = 9259, scaffold N50 = 835,301; contig N50 = 20,803; [50]) using the RNAseq aligner STAR with default parameters (v. 2.5 [51]). We used the featureCounts function of the Rsubread package [52] requiring paired‐end and reverse stranded options to generate read counts across 24,952 previously annotated features [50] with an average coverage depth of 136 reads (S2 and S3 Tables). We assessed mapping and count quality using MultiQC [53]. We previously showed that there was no difference between generalists and molluscivores in the proportion of reads that map to annotated features of the *Cyprinodon* reference genome [49]. Similarly, here we found no difference in the proportion of reads mapping to features between generalists, molluscivores, and hybrids (S2 Fig; ANOVA, *P* = 0.6), but we did find that fewer reads mapped to features in 17–20 dpf samples than 8 dpf samples (ANOVA, *P* = 2.38 × 10^−10^).

Since we analyzed RNA from 43 individuals that were sequenced across four different dates and their libraries were prepared with either KAPA or TruSeq stranded mRNAseq kits, we tested whether a significant amount of between-sample variance in read counts was explained by sequencing date or library preparation kit. We fit linear models (using the lm() function in R) to determine whether normalized counts across individuals were influenced by 1) the number of duplicate reads, 2) the uniformity of coverage across a transcript, or 3) the depth of coverage across GC-rich transcripts. All of these measures could have been influenced by different library preparation methods [54–56]. RseQC identified duplicates as paired reads that mapped to the exact same locations. These can be natural duplicates (and informative for differential expression comparisons) or result from differences in fragmenting and PCR amplification methods used by different library preparation kits [57]. We quantified the number of duplicate reads and the median percent GC content of mapped reads for each sample using RSeQC [58]. We also used RSeQC to estimate transcript integrity numbers (TINs) which is a measure of potential *in vitro* RNA degradation within a sample. TIN is calculated by analyzing the uniformity of coverage across transcripts [58,59]. We performed ANOVA to determine whether the proportion of duplicate reads, GC content of reads, TINs, the number of normalized read counts, number of raw read counts, or number of raw fastq reads differed between samples grouped by library preparation method and by sequencing date.

### Differential expression analyses and hybrid inheritance of expression patterns

We performed differential expression analyses with DESeq2 (v. 3.5 [60]). This program fits negative binomial generalized linear models for each gene across samples to test the null hypothesis that the fold change in gene expression between two groups is zero. DESeq2 uses an empirical Bayes shrinkage method to determine gene dispersion parameters, which models within-group variability in gene expression, and logarithmic fold changes in gene expression. DESeq2 normalizes raw read counts by calculating a geometric mean of counts for each gene across samples, dividing individual gene counts by this mean, and using the median of these ratios as a size factor for each sample. These sample-specific size factors account for differences in library size and sequencing depth between samples. Gene features showing less than 10 normalized counts in every sample in each comparison were discarded from analyses. These filtering criteria would exclude genes that are only expressed in one group. However, this conservative threshold discarded those genes that showed low coverage across all samples, which would have low power to detect differences in expression between groups. Differential expression between groups was determined with Wald tests by comparing normalized posterior log fold change estimates and correcting for multiple testing using the Benjamini–Hochberg procedure with a false discovery rate of 0.05 [61]. We also used DESeq2 to perform clustering and principal component analyses (S3 Fig).

We conducted pairwise comparisons to identify genes differentially expressed between hybrids vs. parental species, hybrids vs. generalists, hybrids vs. molluscivores, and generalists vs. molluscivores. “Parental species” refers to generalists and molluscivores derived from the same populations as the parents of the hybrid samples. We did not sequence any of the parents crossed to generate hybrids. We defined genes as misregulated in hybrids if they were significantly differentially expressed between hybrids and the parental species samples. First, we compared whole-larvae gene expression between samples collected at 8 dpf (six generalists, six molluscivores, and three hybrids). All of the 8 dpf samples were sequenced at the Vincent J. Coates Genomic Sequencing Laboratory, University of California Berkeley (VJCGSL UCB) and their libraries were all prepared using the TruSeq stranded mRNAseq kit. Second, we compared craniofacial tissue gene expression between samples collected at 17–20 dpf (six generalists, six molluscivores, and four hybrids). The generalist and molluscivore samples were sequenced at the High-Throughout Sequencing Facility, University of North Carolina Chapel Hill (HTSF UNC) and their libraries were prepared using the KAPA stranded mRNA-seq kit, while the hybrids were sequenced at the VJCGSL UCB and their libraries were prepared using the TruSeq kit. In order to understand how sequencing at different facilities and using different library prep methods affected the proportion of genes misregulated between hybrids and parental species at 17–20 dpf, we performed a third set of comparisons between hybrids collected at 8 dpf (sequenced at VJCGSL UCB with TruSeq) and generalists and molluscivores from a previous study collected at 8–10 dpf (sequenced at HTSF UNC with KAPA; [49]). We measured how many genes were differentially expressed between 8 dpf hybrids vs. 8–10 dpf parental species than there were differentially expressed between 8 dpf hybrids vs. 8 dpf parental species. This allowed us to estimate an upper-limit on the proportion of genes falsely identified as differentially expressed between 17–20 dpf hybrids and 17–20 dpf parental species due to samples being sequenced at different facilities with different library preparation kits.

**Fig 2:**
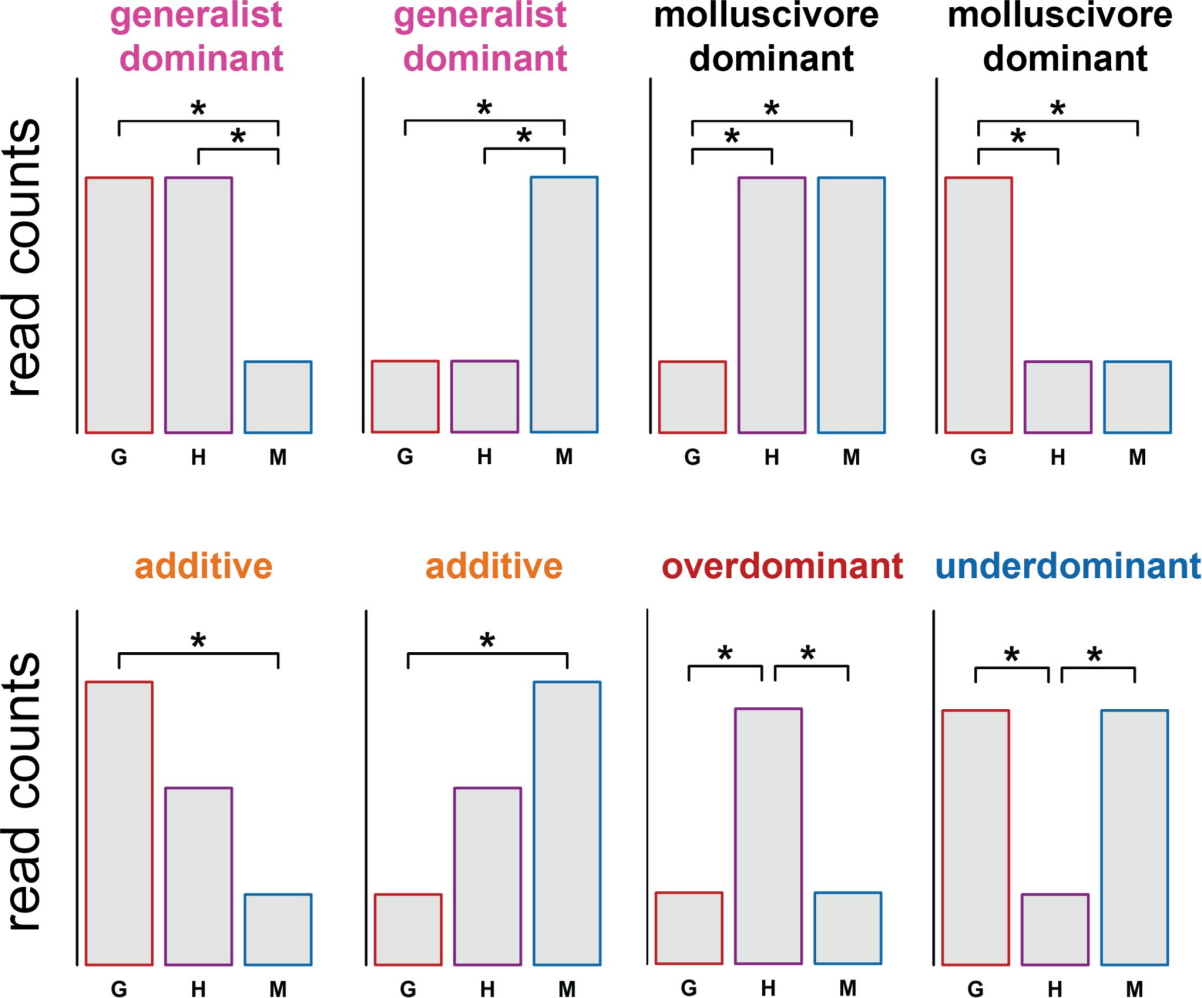
Classifying gene expression inheritance in hybrids. Schematic showing how gene expression inheritance in hybrids was classified. Asterisks indicate significant differential expression between groups. G = generalists, H = F1 hybrids, M = molluscivores.

To determine whether genes showed additive, dominant, or transgressive patterns of inheritance, we quantified differences in gene expression between hybrids vs. parental species and compared them to genes differentially expressed between generalists vs. molluscivores (Fig 2). Hybrid inheritance was considered additive if hybrid gene expression was intermediate between generalists and molluscivores with significant differential expression between generalists and molluscivores, respectively. Inheritance was dominant if hybrid expression was significantly different from one parent species but not the other. Genes showing misregulation in hybrids showed transgressive inheritance, meaning hybrid gene expression was significantly higher (overdominant) or lower (underdominant) than both parental species.

### Gene ontology enrichment analyses

The *Cyprinodon* reference genome is annotated for genomic features (NCBI, *C*. *variegatus* Annotation Release 100, [50]), and many annotated genes share the same name as their zebrafish orthologs. Of the 26,522 protein coding genes annotated for the *Danio rerio* GRCz11 genome annotation release 106 and the 23,373 protein coding genes annotated for the *Cyprinodon* reference genome, 7,222 genes share the same name. We performed gene ontology (GO) enrichment analyses for genes differentially expressed between species and misregulated in hybrids that shared the same name as zebrafish orthologs using GO Consortium resources available at geneontology.org [62]. We searched for enrichment across biological process ontologies curated for zebrafish.

### Allele specific expression and mechanisms of regulatory divergence

We followed the best practices guide recommended by the Genome Analysis Toolkit (v. 3.5 [63]) in order to call and refine SNP variants within coding gene regions using the Haplotype Caller function. We called SNPs across all filtered reads mapped to annotated features for 17–20 dpf samples and 8 dpf samples using conservative hard-filtering parameters [63]: Phred-scaled variant confidence divided by the depth of nonreference samples > 2.0, Phred-scaled *P*-value using Fisher's exact test to detect strand bias > 60, Mann–Whitney rank-sum test for mapping qualities (z > 12.5), Mann–Whitney rank-sum test for distance from the end of a read for those with the alternate allele (z > 8.0). We used the VariantsToTable function (with genotypeFilterExpression "isHet = = 1") to output heterozygous variants for each individual. We counted the number of reads covering heterozygous sites using the ASEReadCounter (with -U ALLOW_N_CIGAR_READS -minDepth 20—minMappingQuality 10—minBaseQuality 20 -drf DuplicateRead). In total we identified 15,429 heterozygous sites across all 32 individuals with sequencing coverage ≥ 20× that fell within 3,974 genes used for differential expression analyses. At the 8 dpf stage, we found 2,909 of the 3,974 genes that contained heterozygous sites common to all samples. At the 17–20 dpf stage, we found 2,403 genes containing heterozygous sites common to all samples.

We assigned each heterozygous allele as the reference allele, alternate allele, or second alternate allele and matched each allele to its corresponding read depth. This allowed us to identify allele specific expression (ASE) by measuring expression variation between the two sites. We only measured ASE at sites that were heterozygous in all samples in each stage in order to account for differences in nucleotide diversity within populations [47]. We used a binomial test in R (binom.test) to determine if a heterozygous site showed significantly biased expression of one allele over another (*P* < 0.05; [23,24]). We measured ASE across 2,909 genes that contained heterozygous sites common to all 8 dpf samples and 2,403 genes that contained heterozygous sites common to all 17–20 dpf samples. A gene was considered to show ASE in hybrids if a heterozygous SNP within that gene showed consistent biased expression in all hybrid samples (17–20 dpf n = 4; 8 dpf n = 3) and did not show ASE in the parental samples (n = 12 for both developmental stages). We also estimated a more conservative measure of ASE at the gene level using MBASED [64], which uses a pseudo-phasing approach that assigns an allele with a larger read count at each SNP to the 'major' haplotype, assuming that ASE is consistent in one direction along the length of the gene. This program calculates ASE using a beta-binomial test comparing the counts of alternate alleles across each gene. For each sample, we performed a 1-sample analysis with unphased gene counts using default parameters run for 1,000,000 simulations to identify genes showing significant ASE (P < 0.05).

A common approach to identify regulatory mechanisms underlying expression divergence is to measure ASE at sites that are heterozygous in hybrids and alternately homozygous in parental species [22,25]. However, generalists and molluscivores diverged recently and there are no fixed SNPs within coding regions out of a total of over 12 million variants examined in 42 resequenced genomes [49]. We measured ASE across heterozygous sites in parental populations to exclude genes which already showed ASE in a pure species background and then determined which genes showed ASE unique to hybrids to make inferences about putative compensatory divergence underlying hybrid misregulation. Gene expression controlled by compensatory variation in parental species is often associated with misregulation in their hybrids [7,31–33]. Regulatory elements that have opposite effects on the expression level of a particular gene can compensate for one another to produce an optimal level of gene expression favored by stabilizing selection [30,31]. Diverging species can evolve alternate compensatory mechanisms while maintaining similar expression levels [65]. Hybrids of such species would have a mismatched combination of regulatory elements that no longer compensate one another, which is expected to result in biased expression of parental alleles [22,32]. Thus, we identified gene expression controlled by putative compensatory regulatory variation if a gene 1) did not show differential expression between generalists and molluscivores, 2) showed significant ASE at one or more heterozygous sites in F1 hybrids, and 3) did not show ASE at any site in purebred generalists or molluscivores. Finally, we looked for overlap between genes showing compensatory regulation and genes showing misregulation in hybrids.

## Results

### Differential expression between generalists and molluscivores

We previously found 1,014 genes differentially expressed in whole-larvae tissue between six generalists and six molluscivores collected 8–10 days post fertilization (dpf; [49]). Here we compared gene expression in whole-larvae tissue collected at exactly 8 dpf (190–194 hours after fertilization rather than 8–10 dpf) between six generalists and six molluscivores. We found 700 out of 17,723 (3.9%) genes differentially expressed between species (Fig 1C). 235 of the 700 genes were annotated as zebrafish orthologs and used for gene ontology enrichment analyses. Encouragingly, the only significantly overrepresented ontology was skeletal system morphogenesis (GO:0048705) which matched 11 differentially expressed genes (S4 Table).

We previously found 120 genes differentially expressed in craniofacial tissue between species at 17–20 dpf [49]. Here we reexamined gene expression in those same individuals using a more conservative threshold for genes to be included in differential expression analyses (where a gene must show > = 10 normalized counts in every sample included in the comparison). As expected, we found fewer genes differentially expressed using this more conservative threshold (81 out of 13,901 (0.6%); Fig 1E). These 81 genes did not show enrichment for any biological process ontologies.

### Hybrid misregulation in whole-larvae tissue

**Fig 3:**
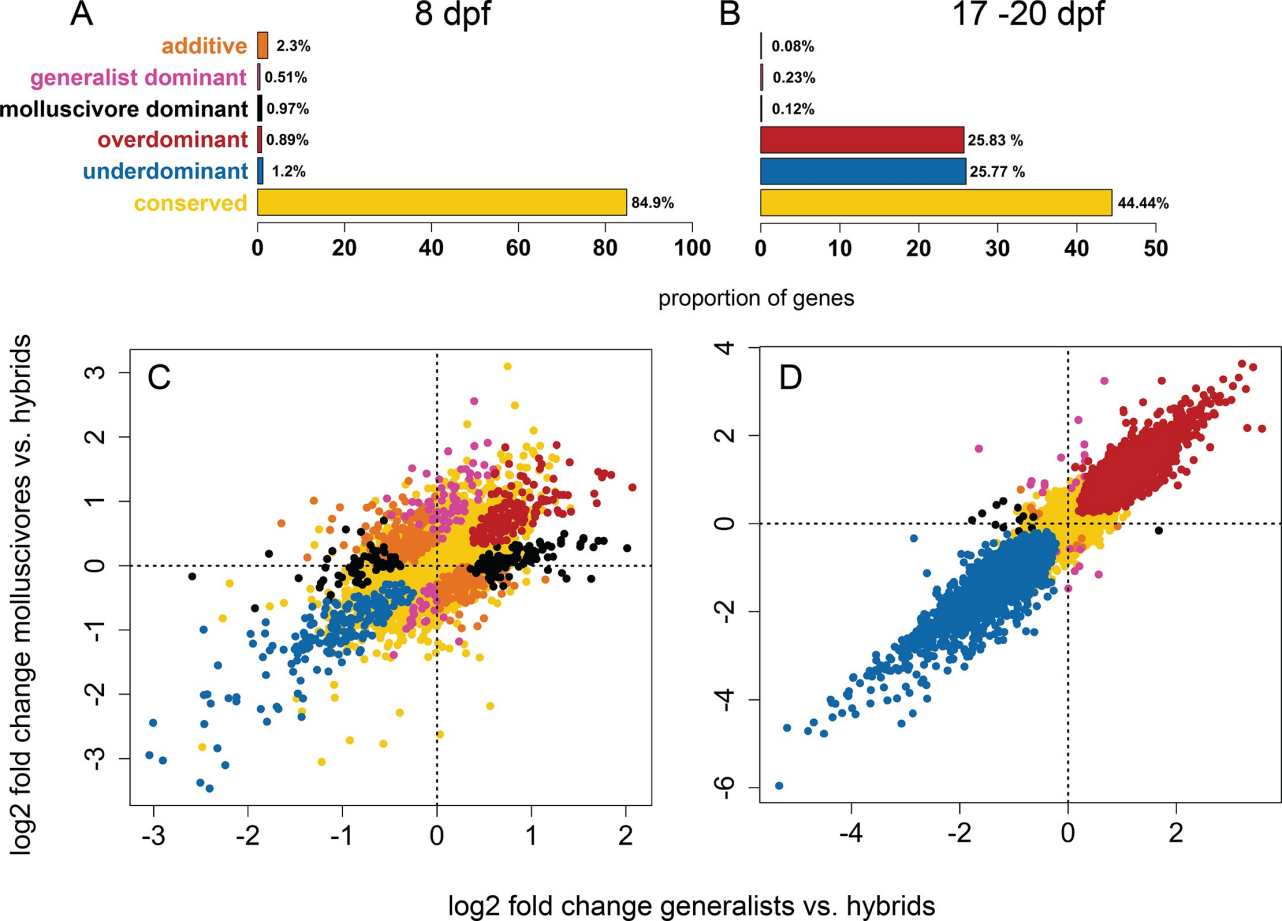
Gene expression inheritance in hybrids. The proportion of (A) 17,705 and (B) 12,769 genes showing each class of hybrid gene expression inheritance. Log2 fold changes in gene expression between molluscivores vs. hybrids on the y-axis and between generalists vs. hybrids on the x-axis for (C) whole-larvae sampled 8 days post fertilization (dpf) and (D) craniofacial tissues dissected from 17–20 dpf samples.

We compared gene expression in whole-larvae tissue collected at 8 dpf from generalist and molluscivore populations (n = 12) with expression in their F1 hybrids (n = 3) and found that 370 out of 17,705 genes (2.1%) were misregulated in hybrids (Fig 1D). Slightly more genes showed underdominant inheritance (209; 1.2%) than overdominant inheritance (154; 0.89%; Fig 3A and 3C). The magnitude of differential expression was higher for genes showing underdominant inheritance than overdominant inheritance (S4 Fig; Wilcoxon rank sum test, *P* = 8.5 × 10^−5^). Of the 370 genes showing misregulation, 138 were annotated as zebrafish orthologs used for gene ontology enrichment analyses. The only significantly overrepresented term was cellular lipid metabolic process (GO:0044255).

The majority of genes showed conserved levels of expression with no significant difference between hybrids and parental species (84.9%). In line with other hybrid expression studies [19,27,28], most genes that did not show conserved inheritance showed additive inheritance (399; 2.3%). We found some genes showing evidence for dominance, with 89 (0.51%) showing ‘generalist-like’ expression patterns and 168 (0.97%) showing ‘molluscivore-like’ patterns of inheritance (Fig 3A and 3C).

### Hybrid misregulation in craniofacial tissue

We compared gene expression in craniofacial tissue collected at 17–20 dpf from generalist and molluscivore populations (n = 12) with expression in their F1 hybrids (n = 4) and found extensive hybrid misregulation. More than half of genes (6,590 out of 12,769 (51.6%)) were differentially expressed in hybrids compared to parental species expression (Fig 1F). There was an approximately equal number of genes showing overdominant and underdominant expression in hybrids, with 3,299 (25.83%) genes showing higher expression in hybrids relative to parental species and 3,291 (25.77%) showing lower expression in hybrids (Fig 1F, Fig 3B and 3D). While there was a similar number of genes showing over- and underdominance, the magnitude of differential expression was higher for genes showing underdominance (S4 Fig; Wilcoxon rank sum test, *P* < 2.2 × 10^−16^). Of the 6,590 genes showing misregulation, 2,876 were annotated as zebrafish orthologs used for gene ontology enrichment analyses. Misregulated genes were enriched for 210 ontologies, including embryonic cranial skeleton morphogenesis (GO:0048701; S5 and S6 Tables).

### Hybrid misregulation is influenced by library preparation and sequencing conditions

All of the 8 dpf samples were sequenced at the same facility using the same library preparation kit. However, the 17–20 dpf generalist and molluscivore samples were sequenced at a different facility than the 17–20 dpf hybrid samples and used a different library preparation kit. We took two approaches toward understanding how sequencing at different facilities and using different library kits may have affected the proportion of genes misregulated between hybrids and parental species at 17–20 dpf.

First, we performed another differential expression comparison between whole-larvae hybrids collected at 8 dpf and whole-larvae parental species that we collected for a previous study between 8–10 dpf [49]. The 8 dpf hybrids were sequenced at the same facility with the same library kit as the 17–20 dpf hybrids, while the 8–10 dpf parental species were sequenced at the same facility with the same library kit as the 17–20 dpf parental species. This design mirrored the comparison we used to estimate 17–20 dpf hybrid craniofacial misregulation, but at an earlier developmental stage (S5 Fig). Whereas comparisons between 8 dpf hybrids and parental species sequenced under the same conditions revealed 370 genes (2.1%) misregulated, comparisons between hybrids and parental species sequenced at different sequencing centers with different library preparation kits suggested that 997 (6%) genes were misregulated–a 37% increase (S5 Fig). This presents a major caveat to our findings, but does not suggest that all genes showing hybrid misregulation in 17–20 dpf craniofacial tissues are false-positives. Using this estimate of bias to correct for different library preparation methods for our 17–20 dpf samples, we estimate that 19.1% genes were misregulated in hybrid craniofacial tissue rather than the raw estimate of 51.6%.

**Fig 4:**
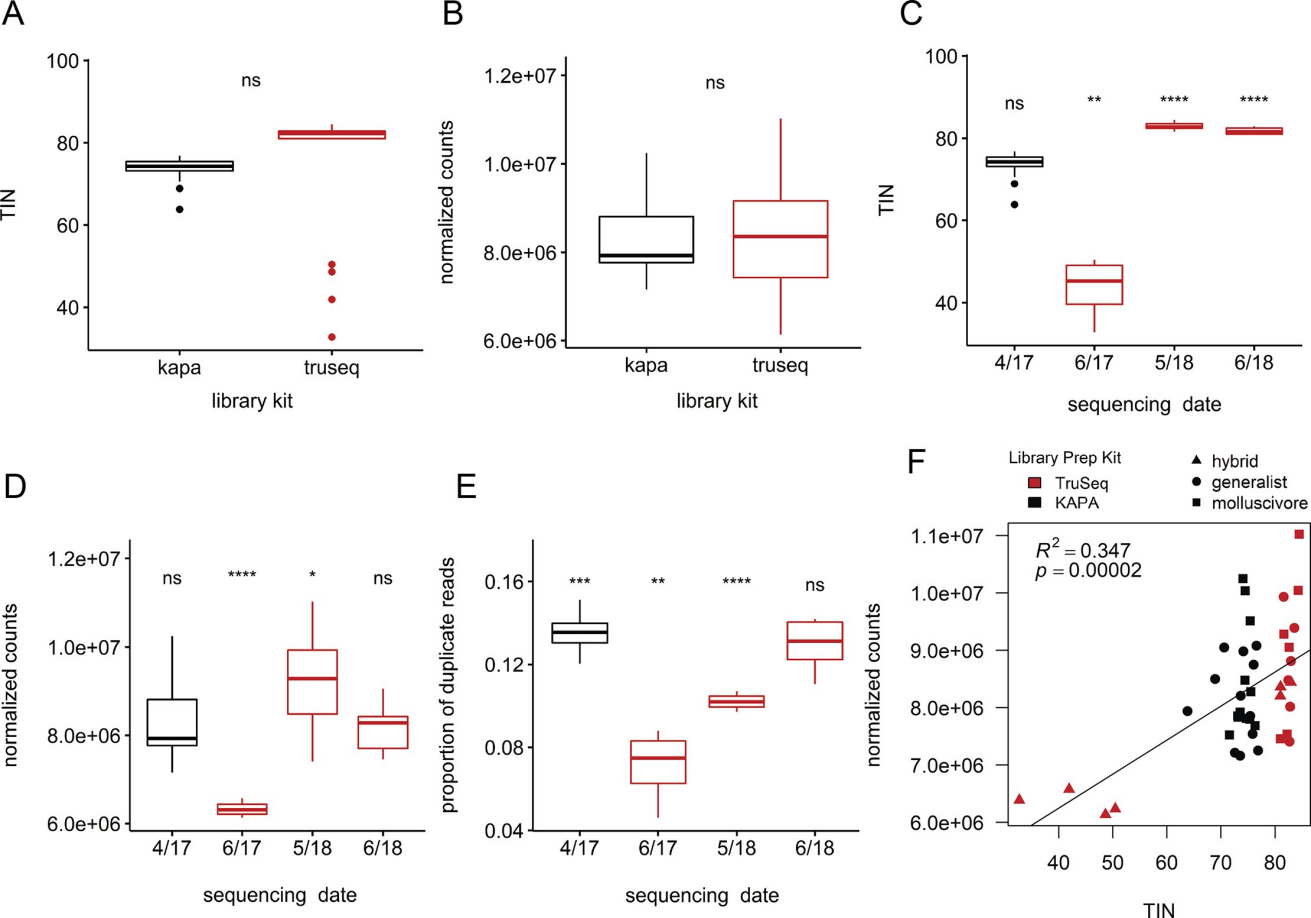
Effects of sequencing facility and library preparation kit. Boxplots show samples grouped by library preparation method (A and B) or by the date they were sequenced (C-E) and whether samples were prepared using Truseq stranded mRNA library kits (red) or KAPA stranded mRNA library kits (black). There was no difference in (A) median transcript integrity numbers (TIN) or (B) number of normalized counts between groups prepared with different library kits (Welch two sample t-test, *P* > 0.05). Hybrid craniofacial sampled 17–20 days post fertilization (sequenced 6/17) showed significantly lower (C) TIN, (D) normalized read counts, and (D) proportion of duplicate reads compared to samples sequenced on other dates (Pairwise Welch two sample t-test; *P* < 0.0001 = ****, *** = 0.001, ** = 0.01, * = 0.05). F) Lower TIN was correlated with lower normalized read count.

We also investigated whether a significant amount of between-sample variance in read counts was explained by library preparation method or sequencing date. For each sample we quantified the number of normalized read counts, raw read counts, and raw fastq reads. We also estimated the proportion of duplicate reads out of total mapped reads, the median percent GC content across mapped reads, and the uniformity of coverage across mapped reads (median transcript integrity numbers (TINs)). All of these measures could be influenced by different library preparation methods [54–56]. However, library preparation method was not associated with differences in the number of normalized read counts or median TINs (Fig 4A and 4B; Welch two sample t-test, *P* > 0.05). When we grouped samples by sequencing date rather than library preparation method, we found that the 17–20 dpf hybrid craniofacial samples (sequenced 6/17) did not show any difference in median GC content, raw read counts, or raw fastq reads compared to samples sequenced on different dates (S6 Fig). However, these samples did show lower proportions of duplicate reads, fewer normalized read counts, and lower TINs compared to samples sequenced on all other dates (Fig 4C–4E; ANOVA; *P* < 0.01). TINs quantify the uniformity of coverage across transcripts and are informative as a measure of *in vitro* RNA degradation, which likely suggests that hybrid craniofacial samples experienced more degradation than other samples prior to sequencing. Indeed, lower TIN was significantly correlated with a lower number of normalized counts across samples (Fig 4F; linear regression; *P* = 2.0 × 10^−5^). We found approximately the same number of genes overexpressed in hybrids (25.83%) as there were genes underexpressed (25.77%), suggesting that many genes were overexpressed in hybrids despite potential RNA degradation.

Overall, we found that our estimate of the proportion of genes misregulated in 17–20 dpf hybrid craniofacial tissue (51.6%) was biased due to differences in the number of duplicate reads produced by two different library preparation methods (Fig 4E). We quantified this bias by measuring hybrid misregulation between samples collected at an earlier developmental stage and found that 19.1% of genes were misregulated in 17–20 dpf hybrid craniofacial tissues after correcting for library preparation biases (S5 Fig). We found that 17–20 dpf hybrid craniofacial tissues likely experienced more *in vitro* RNA degradation than other samples, but this did not produce a bias toward more genes showing underdominant expression in hybrids (Fig 3B).

### Putative compensatory variation underlies misregulation in hybrids

If a gene shows similar gene expression levels between parental species but shows biased allelic expression only in hybrids, it may be regulated by compensatory variation, and such genes are likely to be misregulated in F1 hybrids [31,32]. We identified 15,429 heterozygous sites across all 8 dpf and 17–20 dpf individuals with sequencing coverage ≥ 20× that fell within 2,909 (8 dpf) and 2,403 (17–20 dpf) genes used for differential expression analyses. We estimated allele specific expression (ASE) for these genes and paired these data with patterns of differential expression between parental species to identify genes controlled by putative compensatory variation.

**Fig 5:**
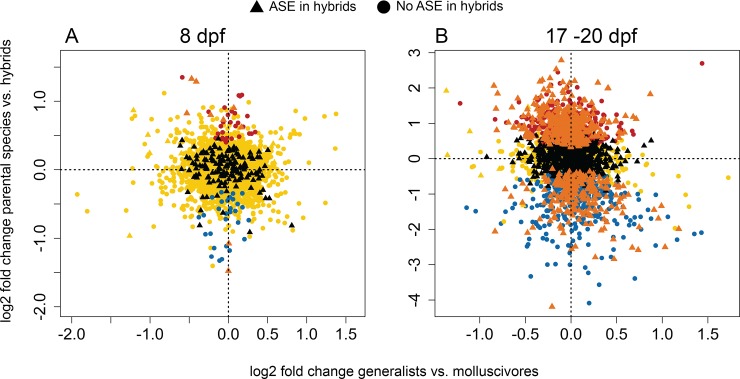
Putative compensatory regulation underlying expression divergence between generalists and molluscivores. Log2 fold changes in gene expression between parental species vs. hybrids on the y-axis and between generalists vs. molluscivores on the x-axis for (A) 2,909 genes containing heterozygous sites used for allele specific expression (ASE) analyses in whole-larvae sampled 8 days post fertilization (dpf) and (B) 2,403 genes containing heterozygous sites in 17–20 dpf craniofacial samples. Triangle points indicate genes showing significant ASE in all hybrids that did not show ASE in generalists or molluscivores. Circle points indicate genes that did not show significant ASE in hybrids or did not show ASE unique to hybrids. Orange = compensatory regulation and hybrid misregulation (genes showing ASE in hybrids, no difference in expression between generalists and molluscivores, and misregulation in hybrids). Black = compensatory regulation (genes showing ASE in hybrids, no difference in expression between generalists and molluscivores). Blue = overdominant (upregulated in hybrids). Red = underdominant (downregulated in hybrids). Yellow = conserved/ambiguous (No difference in expression between parental populations and hybrids).

We measured ASE across sites within 2,770 genes that showed no difference in expression between generalists and molluscivores at 8 dpf. We found 157 genes (5.4%) that were likely regulated by compensatory mechanisms, which showed ASE only in hybrids and were not differentially expressed between generalists and molluscivores. Of these, nine genes (0.33%) also showed misregulation in hybrids (Fig 5A and 5C). We also measured ASE across sites within 2,387 genes that showed no difference in expression between generalists and molluscivores at 17–20 dpf. We found 1080 genes (44.81%) that were likely regulated by compensatory mechanisms. In support of this wide-spread compensatory regulation, 581 of these 1080 genes (53.8%) also showed misregulation in hybrids (Fig 5B and 5D). These 581 genes showed enrichment for protein maturation, mRNA splicing, macromolecule catabolic process, and intracellular catabolic process.

We also found more genes showing compensatory regulation in 17 dpf tissues than 8 dpf tissues using a more conservative method to identify genes showing ASE with MBASED [64]. At 8 dpf, 61 genes (2.2%) showed expression patterns consistent with compensatory regulation, and 18 (0.65%) were misregulated in hybrids. At 17 dpf, 95 genes (3.98%) showed expression patterns consistent with compensatory regulation, and 55 (2.30%) were misregulated in hybrids.

**Fig 6:**
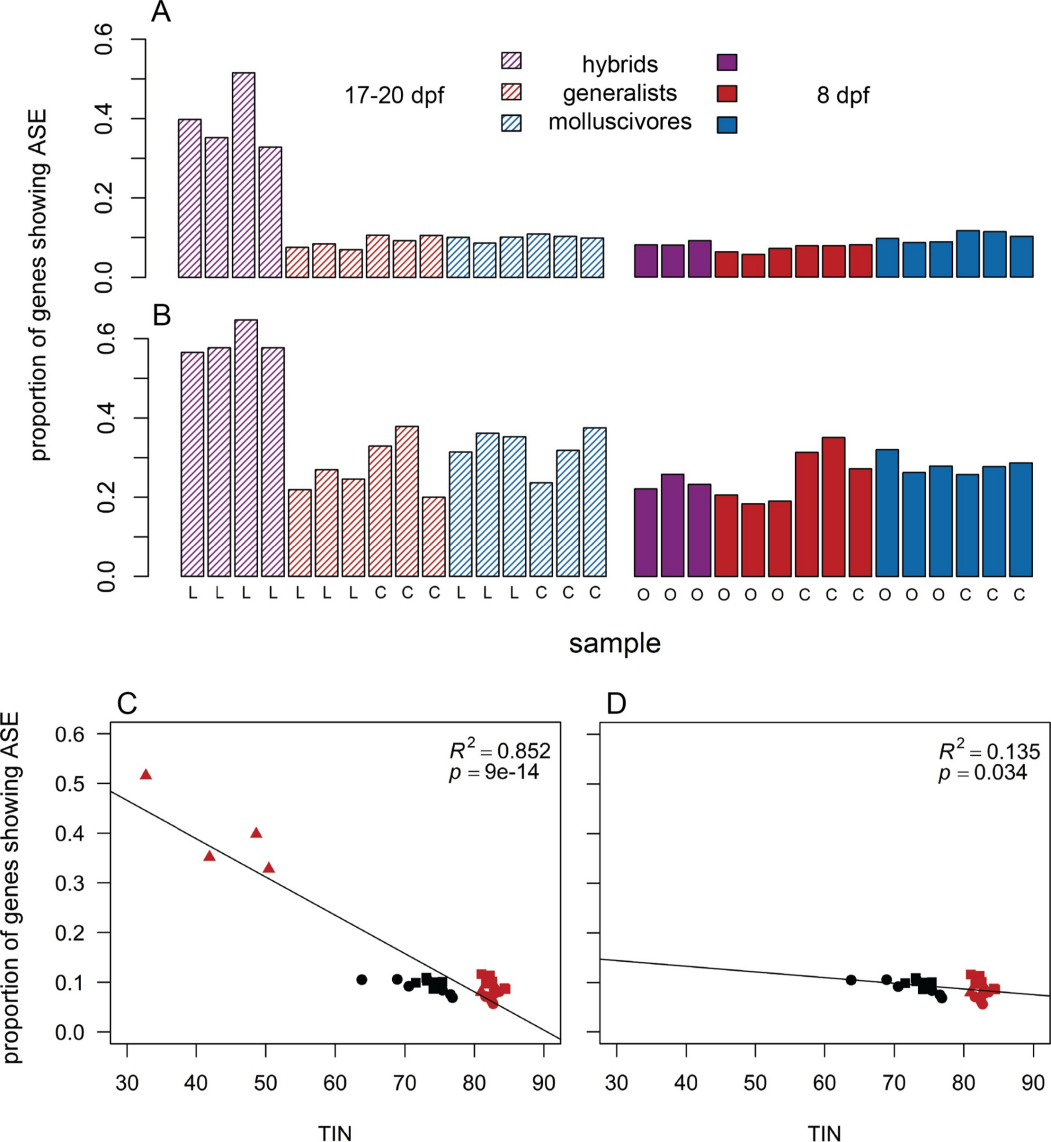
Hybrid craniofacial tissues show high levels of allele specific expression. F1 hybrid craniofacial tissues sampled 17–20 days post fertilization (dpf; striped purple bars) showed a higher proportion of genes showing significant allele specific expression compared to all other samples using a coverage threshold of (A) ≥ 10× reads supporting each heterozygous allele (ANOVA, *P* = 2.81 × 10^−5^) and (B) ≥ 100× reads supporting each allele (ANOVA, *P* = 3.85 × 10^−4^). 8 dpf = solid, 17–20 dpf = striped; hybrids = purple, generalists = red, molluscivores = blue; L = Little Lake, C = Crescent Pond, O = Osprey Lake. (C) TIN was significantly negatively correlated with ASE (linear regression; *P* = 9.04 × 10^−14^). (D) This correlation persisted when 17–20 dpf hybrid craniofacial samples were excluded from the linear model (linear regression; *P* = 0.034). However, the observed proportion of genes showing ASE was much higher in 17–20 dpf hybrid craniofacial samples than predicted by the linear model in (D).

We found many more genes showing ASE (using binomial tests) in 17–20 dpf hybrid craniofacial tissue than any other samples (Fig 6A; ANOVA, *P* = 2.81 × 10^−5^). Since misregulation is expected in hybrids when gene expression is controlled by compensatory variation between parental species [31–33], the high number of genes showing putative compensatory regulation and high number of genes showing ASE in hybrids supports the pattern of extensive misregulation in 17–20 dpf hybrid craniofacial tissue. We likely overestimated the amount of misregulation in this tissue because hybrids were sequenced using a different library preparation kit than parental species (see above). However, ASE was estimated by examining allelic ratios in individual samples. 17–20 dpf hybrid craniofacial tissue was sequenced at the same facility using the same library preparation kit as all of the 8 dpf samples (Table 1 and S1 Table), yet we only found a high number of genes showing ASE in the 17–20 dpf hybrids (Fig 6A).

We tested whether this pattern might be due to higher rates of *in vitro* degradation in hybrid samples (reflected by low TINs), which could increase variance in the abundance of reads at heterozygous sites and bias ASE estimates. Lower TIN was correlated with higher ASE (Fig 6D; linear regression; *P* = 9.04 × 10^−14^). This correlation persisted when 17–20 dpf hybrid craniofacial samples were excluded from the model (Fig 6E; linear regression; *P* = 0.034), suggesting that rates of mRNA degradation may differ depending on genotypes at heterozygous sites. While this explains some of the elevated ASE in 17–20 dpf hybrid craniofacial samples, the proportion of genes showing ASE was much higher in these samples than predicted by the latter linear model. Even the lowest TIN for a 17–20 dpf hybrid sample (32.68) predicted a much lower range of genes showing ASE (8.2% -14.1%) compared to the observed range (32.8% - 51.6%). Finally, we also estimated ASE again with a higher coverage threshold (> = 100 counts supporting each heterozygous allele) to reduce the chances of increased variance affecting binomial tests and still found that hybrid craniofacial samples showed more ASE than other samples (Fig 6B; ANOVA, *P* = 3.85 × 10^−4^).

## Discussion

Molluscivores show extreme craniofacial divergence relative to their generalist sister species, exhibiting a novel maxillary protrusion and short robust jaws (Fig 1A; [34,66]). Given the extreme craniofacial divergence observed between molluscivores and their generalist sister-species, we might expect to find genes expressed in hybrids outside the range of either parent species as a result of discordance between alternatively coadapted genes in regulatory networks shaping divergent craniofacial morphologies. However, genetic divergence between generalists and molluscivores is low, with only 79 SNPs fixed between species (genome-wide average F_st_ = 0.08, D_xy_ = 0.00166; [47,49]). Despite this low genetic divergence and ongoing gene flow between species, we found gene misregulation in F1 hybrids at two developmental stages and tissue types. We also measured allele specific expression (ASE) for genes expressed in hybrids and parental species and found evidence for putative compensatory divergence influencing hybrid misregulation at both developmental stages.

### Hybrid misregulation during juvenile development

While many studies on hybrid misregulation search for regulatory divergence in ‘speciation genes’ associated with sterility and inviability [4,14,19,24], our results highlight the importance of considering misregultion over multiple early developmental stages and in the context of adaptive morphological traits. We found evidence of misregulation in whole-larvae hybrid tissues sampled eight days post fertilization (dpf; 2.1% of genes) and in 17–20 dpf hybrid craniofacial tissues (19.1% of genes after correcting for bias due to library preparation method).

There are several reasons why we might expect to find a higher proportion of genes misregulated in 17–20 dpf hybrid craniofacial tissues relative to 8 dpf whole-larvae tissues. The molluscivore shows exceptional rates of morphological diversification, particularly in craniofacial traits [38]. Perhaps 17–20 dpf is a crucial developmental window when gene networks shaping these traits become extensively misregulated in hybrids. It is just after this stage that the relative length of the premaxilla, maxilla, palatine, and lower jaw tend to increase more for generalists than molluscivores [41]. It is also possible that regulatory changes are compounded throughout development and have cascading effects, resulting in higher rates of misregulation in later stages. Finally, some of the increased misregulation in hybrid craniofacial tissue can likely be attributed to our sampling design. We found that hybrid craniofacial samples showed lower TINs and lower normalized counts (Fig 4A and 4D), suggesting that these samples may have experienced more *in vitro* RNA degradation than other samples [59]. While it is difficult to predict how much overdominance we would expect in these samples given that misregulation has not been previously studied in isolated craniofacial tissues, we found approximately the same number of genes overexpressed in hybrids (25.83%) as there were genes underexpressed (25.77%), suggesting that many genes were overexpressed in hybrids despite potential RNA degradation.

We found roughly twice the amount of bias-corrected misregulation in hybrid craniofacial tissues compared to a study of misregulation in whole-larvae tissue that measured gene expression in F1 hybrids generated between benthic and limnetic lake whitefish [12,67]. These populations also diverged within the past 10 kya and occupy different habitats within lakes [68]. We also found that genes showing underdominance in hybrids showed a higher magnitude of differential expression compared to those showing overdominance in 8 dpf and 17–20 dpf tissues (S4 Fig), a pattern that has also been observed in lake whitefish [67] and a generalist/specialist *Drosophila* species pair [23].

### The consequences of hybrid misregulation

It is unclear whether such extensive gene misregulation in hybrid craniofacial tissues might contribute to intrinsic postzygotic isolation between generalists and molluscivores. F2 hybrids exhibiting intermediate and transgressive craniofacial phenotypes showed reduced survival and growth rates in the wild relative to F2 hybrids resembling parental species [39,46], but short-term experiments measuring F2 hybrid survival in the lab did not find any evidence of reduced survival for hybrids with intermediate phenotypes [46]. This was interpreted as evidence that complex fitness landscapes measured in field enclosures on San Salvador with multiple peaks corresponding to the generalist and molluscivore phenotypes were due to competition and foraging ability in the wild (i.e. extrinsic reproductive isolation). However, additional analyses of these data suggest that absolute performance of hybrids may also play a role in their survival. The most transgressive hybrid phenotypes exhibited the lowest fitness, contrary to expectations from negative frequency-dependent disruptive selection [39]. It is still possible that intrinsic and extrinsic incompatibilities interact such that gene misregulation weakens performance more in the wild than in the lab. However, note that F1 hybrids used in this study exhibit approximately intermediate trophic morphology relative to parental trophic morphology whereas field experiments used F2 and later generation hybrid intercrosses and backcrosses.

### Hybrid misregulation is controlled by putative compensatory divergence

When an optimal level of gene expression is favored by stabilizing selection, compensatory variation can accumulate between species and cause misregulation in hybrids [32,33]. We combined results from differential expression analyses with allele specific expression (ASE) results to identify genes controlled by putative compensatory regulatory divergence between generalists and molluscivores. In 8 dpf whole-larvae tissue, we found 5.4% of genes controlled by compensatory regulation (Fig 5B). The low number of genes controlled by compensatory regulation was reflected by the low number of genes misregulated in 8 dpf hybrids (2.1%). In 17–20 dpf hybrid craniofacial tissues, we found 44.81% of genes controlled by compensatory regulation (Fig 5B). The large number of genes controlled by compensatory regulation is consistent with the extensive misregulation observed in hybrid craniofacial tissue, and the majority of genes showing signs of compensatory regulation were also misregulated in hybrids (53.8%). 17–20 dpf hybrid craniofacial tissue was sequenced at the same facility using the same library preparation kit as the 8 dpf samples, yet we only found a high number of genes showing ASE in the 17–20 dpf hybrids (Fig 6A and 6B). One caveat to this result is that the high levels of ASE estimated using binomial tests were influenced to some extent by RNA degradation (Fig 6C and 6D). To our knowledge, this is the first evidence showing a positive correlation between degradation and ASE, and potential mechanisms underlying differential degradation dependent on heterozygous genotype are unclear. The GC content of mRNAs have been shown to positively correlate with decay rate [69]. Perhaps mRNAs with G and C genotypes are more likely to degrade before their A and T counterparts at heterozygous sites, causing increased ASE in degraded samples. Despite this caveat, linear models showed that degradation did not predict the extremely high levels of ASE found in 17–20 dpf hybrid tissues consistent with high misregulation (Fig 6), although it is unknown whether ASE should increase linearly with degradation over time.

### Conclusion

We found hybrid misregulation in both whole-larvae tissues and craniofacial tissues sampled at early developmental stages. This points to divergent evolution of developmental networks shaping novel traits in the molluscivore. It is unclear whether such misregulation causes intrinsic incompatibilities in hybrids within this recent adaptive radiation. Our results are in line with studies finding widespread compensatory evolution in other systems with greater divergence times between species [7,8,31,70,71]. Investigating mechanisms regulating gene expression between generalists and molluscivores that result in hybrid misregulation will shed light on whether the variants shaping novel traits may also contribute to reproductive isolation. Examining misregulation across multiple early developmental stages in the context of developing tissues that give rise to adaptive traits can paint a more complete picture of genetic incompatibilities that distinguish species.

## Supporting information

S1 Table mRNA sequencing design. (DOCX)

S2 Table Read statistics for samples. (DOCX)

S3 Table Quality control statistics for samples. (DOCX)

S4 Table Differentially expressed genes annotated for effects on skeletal system morphogenesis. Skeletal system morphogenesis (GO:0048705) was the only enriched biological process for genes differentially expressed between generalists and molluscivores at 8 dpf (*P* < 0.05; geneontology.org).
(DOCX)

S5 Table Misregulated genes annotated for effects on embryonic cranial skeleton morphogenesis. Embryonic cranial skeleton morphogenesis (GO:0048701) was one of 210 enriched biological processes for 6,590 genes differentially expressed between hybrids and parental species in craniofacial tissue collected at 17–20 dpf (*P* < 0.05; geneontology.org).
(DOCX)

S6 Table Gene ontologies enriched for 6,590 genes misregulated between hybrids and parental species in 17–20 dpf craniofacial tissues. (DOCX)

S1 Fig **20 day old generalist (top) and molluscivore (bottom)**.
(PDF)

S2 Fig Proportion of reads assigned to features. Proportion of reads assigned to features (yellow), unassigned due to multi-mapping (red), and unassigned due to no match to annotated features (blue) using STAR aligner.
(PDF)

S3 Fig Principal components. The first and second principal component axes accounting for a combined 75% of the total variation between generalist (red), molluscivore (blue), and hybrid (purple) samples across reads mapped to annotated features. Point shape indicates the sequencing date of the sample.
(PDF)

S4 Fig Magnitude of misregulation. Genes showing underdominant expression in hybrids show a higher magnitude of misregulation than genes showing overdominance (Wilcoxon rank sum test; 8 dpf *P* = 8.5 × 10^−5^ 17–20 dpf *P* < 2.2 × 10^−16^).
(PDF)

S5 Fig Estimating the effect of sequencing design on the proportion of genes misregulated in hybrids. The 8 dpf hybrids were sequenced at the same facility with the same library kit as the 17–20 dpf hybrids, while the 8–10 dpf parental species were sequenced at the same facility with the same library kit as the 17–20 dpf parental species. (A) The comparison between 8 dpf parental species and 8 dpf hybrids revealed 370 genes (2.1%) misregulated. (B) The comparison between 8 dpf hybrids and 8–10 dpf parental species revealed 997 (6%) genes misregulated–a 37% increase. We used this inflated estimate to adjust our estimate of misregulation in 17–20 dpf hybrid craniofacial tissues. Red points indicate genes detected as differentially expressed at 5% false discovery rate with Benjamini-Hochberg multiple testing adjustment. Grey points indicate genes showing no significant difference in expression between groups.
(PDF)

S6 Fig GC content, normalized read counts, and raw read counts. We did not find significant differences between 17–20 dpf hybrid craniofacial samples and samples sequenced on other dates for (A) median percent GC content across reads, (B) number of normalized read counts, or (C) number of raw fastq reads. the proportion of duplicate reads for each sample (Pairwise Welch two sample t test; *P* < 0.0001 = ****, *** = 0.001, ** = 0.01, * = 0.05).
(PDF)

S1 Appendix Coding supplement. Examples of command line arguments and DESeq2 scripts for bioinformatics pipelines.
(DOCX)
