# Supplementary material for: Hybrid gene misregulation in multiple developing tissues within a recent adaptive radiation of Cyprinodon pupfishes
Source: PLoS One. 2019 Jul 10;14(7):e0218899. doi: 10.1371/journal.pone.0218899 (PMC6619667; doi:10.1371/journal.pone.0218899)
Supplement: S1 Table — (DOCX) [file pone.0218899.s001.docx]

**Table S1.** mRNA sequencing design.

| round | sequencing date | pooled across *n* lanes | library prep kit |
| --- | --- | --- | --- |
| 1 | 4/17 | 1 | KAPA stranded mRNA |
| 2 | 6/17 | 1 | TruSeq stranded mRNA |
| 3 | 5/18 | 1 | TruSeq stranded mRNA |
| 4 | 7/18 | 3 | TruSeq stranded mRNA |
