# Supplementary material for: Hybrid gene misregulation in multiple developing tissues within a recent adaptive radiation of Cyprinodon pupfishes
Source: PLoS One. 2019 Jul 10;14(7):e0218899. doi: 10.1371/journal.pone.0218899 (PMC6619667; doi:10.1371/journal.pone.0218899)
Supplement: S2 Table — (DOCX) [file pone.0218899.s002.docx]

**Table S2.** Read statistics for samples.

| sample | species | stage | sequencing round | library prep kit | raw fastq reads | reads mapped | raw counts | normalized counts |
| --- | --- | --- | --- | --- | --- | --- | --- | --- |
| 1 | hybrid | 17-20dpf | 2 | truseq | 41912228 | 39531780 | 14471030 | 6134242 |
| 2 | hybrid | 17-20dpf | 2 | truseq | 18451756 | 17360214 | 6363816 | 6577234 |
| 3 | hybrid | 17-20dpf | 2 | truseq | 33541230 | 31461875 | 11473464 | 6386857 |
| 4 | hybrid | 17-20dpf | 2 | truseq | 27720328 | 26006609 | 9659367 | 6234856 |
| 5 | generalist | 8dpf | 3 | truseq | 25656702 | 24407630 | 10461159 | 8815459 |
| 6 | generalist | 8dpf | 3 | truseq | 22804982 | 21721330 | 9245634 | 8017330 |
| 7 | generalist | 8dpf | 3 | truseq | 26313696 | 25476498 | 10757268 | 9390970 |
| 8 | molluscivore | 8dpf | 4 | truseq | 38287748 | 36204014 | 15203504 | 7457667 |
| 9 | molluscivore | 8dpf | 4 | truseq | 34288848 | 32578838 | 13434770 | 7536493 |
| 10 | molluscivore | 8dpf | 4 | truseq | 33962768 | 32384092 | 13443902 | 9051218 |
| 11 | generalist | 8-10dpf | 1 | kapa | 23172714 | 17847934 | 6880522 | 7213838 |
| 12 | generalist | 8-10dpf | 1 | kapa | 20575374 | 19452261 | 7933158 | 8206676 |
| 13 | generalist | 8-10dpf | 1 | kapa | 20631366 | 19202893 | 7750909 | 7161844 |
| 14 | generalist | 17-20dpf | 1 | kapa | 20743782 | 18496992 | 6837539 | 8501245 |
| 15 | generalist | 17-20dpf | 1 | kapa | 18728520 | 16361277 | 6040194 | 9051398 |
| 16 | generalist | 17-20dpf | 1 | kapa | 21338994 | 19399691 | 6922698 | 7940092 |
| 17 | molluscivore | 8-10dpf | 1 | kapa | 19100066 | 17430230 | 6789911 | 7921670 |
| 18 | molluscivore | 8-10dpf | 1 | kapa | 19479052 | 17376013 | 6715924 | 7812869 |
| 19 | molluscivore | 8-10dpf | 1 | kapa | 23224142 | 21581058 | 8519810 | 8476166 |
| 20 | molluscivore | 17-20dpf | 1 | kapa | 21012680 | 18765633 | 7182366 | 7853844 |
| 21 | molluscivore | 17-20dpf | 1 | kapa | 20996520 | 19096064 | 7507215 | 7831725 |
| 22 | molluscivore | 17-20dpf | 1 | kapa | 20731964 | 17371497 | 6216825 | 7522140 |
| 23 | generalist | 8-10dpf | 1 | kapa | 26283022 | 23001257 | 8649498 | 8749038 |
| 24 | generalist | 8-10dpf | 1 | kapa | 29483942 | 27652542 | 11273682 | 7540908 |
| 25 | generalist | 8-10dpf | 1 | kapa | 26094366 | 22722751 | 8639044 | 8982363 |
| 26 | generalist | 17-20dpf | 1 | kapa | 23539660 | 21193066 | 8288540 | 9080255 |
| 27 | generalist | 17-20dpf | 1 | kapa | 22989146 | 20041508 | 7630051 | 7855706 |
| 28 | generalist | 17-20dpf | 1 | kapa | 24875424 | 21412781 | 7819750 | 7254103 |
| 29 | molluscivore | 8-10dpf | 1 | kapa | 25828344 | 22266723 | 8306859 | 7798548 |
| 30 | molluscivore | 8-10dpf | 1 | kapa | 25463686 | 22026757 | 7881773 | 7685499 |
| 31 | molluscivore | 8-10dpf | 1 | kapa | 24912808 | 21994615 | 8135992 | 8278350 |
| 32 | molluscivore | 17-20dpf | 1 | kapa | 24703694 | 21871287 | 8360480 | 10038049 |
| 33 | molluscivore | 17-20dpf | 1 | kapa | 21852694 | 18695831 | 6892571 | 10248139 |
| 34 | molluscivore | 17-20dpf | 1 | kapa | 22560226 | 19029742 | 6997928 | 9514273 |
| 35 | generalist | 8dpf | 3 | truseq | 25934770 | 24751899 | 10559980 | 8480276 |
| 36 | generalist | 8dpf | 3 | truseq | 24781078 | 23652972 | 10100401 | 7407245 |
| 37 | generalist | 8dpf | 3 | truseq | 23199342 | 22179263 | 9546582 | 9933070 |
| 38 | molluscivore | 8dpf | 3 | truseq | 25699038 | 24831966 | 10647567 | 9278752 |
| 39 | molluscivore | 8dpf | 3 | truseq | 31456730 | 30017500 | 12699299 | 11025018 |
| 40 | molluscivore | 8dpf | 3 | truseq | 27239292 | 26189352 | 11032426 | 10043336 |
| 41 | hybrid | 8dpf | 4 | truseq | 27989988 | 26774703 | 11506670 | 8202298 |
| 42 | hybrid | 8dpf | 4 | truseq | 26341200 | 25153435 | 10759990 | 8361452 |
| 43 | hybrid | 8dpf | 4 | truseq | 41450864 | 39962577 | 16950855 | 8441280 |
