# Supplementary material for: Hybrid gene misregulation in multiple developing tissues within a recent adaptive radiation of Cyprinodon pupfishes
Source: PLoS One. 2019 Jul 10;14(7):e0218899. doi: 10.1371/journal.pone.0218899 (PMC6619667; doi:10.1371/journal.pone.0218899)
Supplement: S3 Table — (DOCX) [file pone.0218899.s003.docx]

**Table S3.** Quality control statistics for samples.

| sample | species | stage | median TIN | average depth across features | proportion of duplicate reads | median GC content across reads |
| --- | --- | --- | --- | --- | --- | --- |
| 1 | hybrid | 17-20dpf | 48.63 | 169.59 | 6.80 | 46.42 |
| 2 | hybrid | 17-20dpf | 41.90 | 127.99 | 8.15 | 46.50 |
| 3 | hybrid | 17-20dpf | 32.68 | 165.40 | 4.61 | 45.74 |
| 4 | hybrid | 17-20dpf | 50.43 | 138.23 | 8.79 | 45.99 |
| 5 | generalist | 8dpf | 82.94 | 131.20 | 10.19 | 46.82 |
| 6 | generalist | 8dpf | 82.77 | 129.37 | 10.46 | 47.14 |
| 7 | generalist | 8dpf | 83.55 | 125.86 | 10.03 | 47.30 |
| 8 | molluscivore | 8dpf | 81.01 | 139.18 | 14.19 | 46.22 |
| 9 | molluscivore | 8dpf | 82.25 | 128.50 | 14.18 | 46.91 |
| 10 | molluscivore | 8dpf | 82.59 | 125.39 | 13.67 | 48.03 |
| 11 | generalist | 8-10dpf | 72.56 | 157.17 | 13.08 | 46.25 |
| 12 | generalist | 8-10dpf | 73.65 | 145.73 | 13.00 | 45.42 |
| 13 | generalist | 8-10dpf | 73.53 | 140.65 | 13.40 | 46.28 |
| 14 | generalist | 17-20dpf | 68.89 | 144.59 | 13.97 | 45.36 |
| 15 | generalist | 17-20dpf | 70.57 | 134.99 | 14.22 | 46.27 |
| 16 | generalist | 17-20dpf | 63.81 | 155.01 | 13.60 | 44.83 |
| 17 | molluscivore | 8-10dpf | 73.53 | 132.25 | 13.88 | 46.28 |
| 18 | molluscivore | 8-10dpf | 74.69 | 125.74 | 14.05 | 46.78 |
| 19 | molluscivore | 8-10dpf | 74.43 | 142.56 | 12.79 | 45.92 |
| 20 | molluscivore | 17-20dpf | 73.09 | 132.20 | 14.22 | 46.03 |
| 21 | molluscivore | 17-20dpf | 73.17 | 128.74 | 15.12 | 46.81 |
| 22 | molluscivore | 17-20dpf | 71.57 | 138.66 | 13.06 | 47.44 |
| 23 | generalist | 8-10dpf | 76.01 | 140.15 | 12.42 | 46.50 |
| 24 | generalist | 8-10dpf | 75.82 | 154.90 | 12.05 | 45.65 |
| 25 | generalist | 8-10dpf | 74.11 | 146.22 | 12.72 | 46.21 |
| 26 | generalist | 17-20dpf | 76.56 | 129.96 | 14.25 | 45.57 |
| 27 | generalist | 17-20dpf | 75.39 | 136.84 | 13.92 | 45.89 |
| 28 | generalist | 17-20dpf | 76.83 | 127.75 | 13.48 | 45.58 |
| 29 | molluscivore | 8-10dpf | 75.34 | 132.93 | 13.50 | 45.96 |
| 30 | molluscivore | 8-10dpf | 76.29 | 130.14 | 12.95 | 46.38 |
| 31 | molluscivore | 8-10dpf | 75.54 | 131.94 | 13.25 | 46.49 |
| 32 | molluscivore | 17-20dpf | 74.48 | 142.25 | 14.33 | 45.64 |
| 33 | molluscivore | 17-20dpf | 74.08 | 138.28 | 13.73 | 45.90 |
| 34 | molluscivore | 17-20dpf | 75.39 | 129.94 | 13.65 | 46.43 |
| 35 | generalist | 8dpf | 82.43 | 132.68 | 9.94 | 47.27 |
| 36 | generalist | 8dpf | 82.69 | 125.78 | 10.59 | 47.47 |
| 37 | generalist | 8dpf | 81.58 | 136.72 | 9.71 | 46.98 |
| 38 | molluscivore | 8dpf | 81.63 | 135.55 | 9.91 | 47.33 |
| 39 | molluscivore | 8dpf | 84.49 | 125.89 | 10.69 | 47.31 |
| 40 | molluscivore | 8dpf | 84.31 | 118.45 | 10.35 | 47.61 |
| 41 | hybrid | 8dpf | 80.98 | 134.41 | 12.59 | 47.33 |
| 42 | hybrid | 8dpf | 81.02 | 130.78 | 12.12 | 46.98 |
| 43 | hybrid | 8dpf | 82.94 | 142.90 | 11.04 | 47.51 |
