# Supplementary material for: Hybrid gene misregulation in multiple developing tissues within a recent adaptive radiation of Cyprinodon pupfishes
Source: PLoS One. 2019 Jul 10;14(7):e0218899. doi: 10.1371/journal.pone.0218899 (PMC6619667; doi:10.1371/journal.pone.0218899)
Supplement: S4 Table — Skeletal system morphogenesis (GO:0048705) was the only enriched biological process for genes differentially expressed between generalists and molluscivores at 8 dpf (P < 0.05; geneontology.org). (DOCX) [file pone.0218899.s004.docx]

**Table S4.** Differentially expressed genes annotated for effects on skeletal system morphogenesis (GO:0048705). This ontology was the only enriched biological process for genes differentially expressed between generalists and molluscivores at 8 dpf (*P* < 0.05; geneontology.org).

| gene symbol | log_2_ fold change | *P* |
| --- | --- | --- |
| *bmp3* | 0.511242 | 0.039704 |
| *chd7* | 0.423654 | 0.047135 |
| *foxe1* | -0.63748 | 0.004896 |
| *gata3* | 0.369094 | 0.043925 |
| *gfpt1* | -0.29543 | 0.039977 |
| *hand2* | 0.639402 | 0.012518 |
| *kat6a* | 0.55044 | 0.000901 |
| *matn1* | 1.144529 | 0.049159 |
| *matn4* | 0.447086 | 0.000203 |
| *mecom* | 0.552098 | 0.023904 |
| *polr1c* | -0.68794 | 0.026325 |
