# Supplementary material for: Hybrid gene misregulation in multiple developing tissues within a recent adaptive radiation of Cyprinodon pupfishes
Source: PLoS One. 2019 Jul 10;14(7):e0218899. doi: 10.1371/journal.pone.0218899 (PMC6619667; doi:10.1371/journal.pone.0218899)
Supplement: S5 Table — Embryonic cranial skeleton morphogenesis (GO:0048701) was one of 210 enriched biological processes for 6,590 genes differentially expressed between hybrids and parental species in craniofacial tissue collected at 17–20 dpf (P < 0.05; geneontology.org). (DOCX) [file pone.0218899.s005.docx]

**Table S5.** Misregulated genes annotated for effects on embryonic cranial skeleton morphogenesis (GO:0048701). This ontology was one of 210 enriched biological processes for 6,590 genes differentially expressed between hybrids and parental species in craniofacial tissue collected at 17-20 dpf (*P* < 0.05; geneontology.org).

| gene symbol | log_2_ fold change | *P* |
| --- | --- | --- |
| *alcam* | 0.610547 | 0.000222 |
| *alx1* | -0.85427 | 0.033755 |
| *bmp3* | 0.583685 | 0.022167 |
| *crispld2* | -0.94461 | 0.000382 |
| *dcaf7* | -0.81576 | 9.25E-09 |
| *egr1* | -1.18846 | 0.039205 |
| *fam20b* | 0.893436 | 3.84E-05 |
| *fgf3* | 0.707254 | 0.026311 |
| *foxe1* | -0.86424 | 0.023926 |
| *fst* | -1.04804 | 0.000342 |
| *gfpt1* | 1.260497 | 1.00E-14 |
| *gnptab* | 0.847555 | 0.000209 |
| *hand2* | -1.56118 | 1.71E-05 |
| *irf6* | -1.14833 | 2.02E-09 |
| *itga8* | -0.48604 | 0.037746 |
| *kat6a* | -1.09119 | 7.80E-05 |
| *kdm6a* | -0.57079 | 0.005865 |
| *kras* | 0.855153 | 2.61E-05 |
| *leo1* | -0.47694 | 0.012396 |
| *mapre2* | 1.453761 | 2.81E-11 |
| *mecom* | -1.08419 | 0.017119 |
| *med12* | -1.45818 | 2.03E-15 |
| *med14* | 0.758638 | 0.000319 |
| *ocrl* | 1.025415 | 4.44E-05 |
| *pak1* | 0.960502 | 0.000228 |
| *pbx4* | 1.852615 | 2.31E-12 |
| *pdgfra* | -0.48287 | 0.048917 |
| *phf8* | 0.493499 | 0.007445 |
| *pitx2* | 0.807376 | 0.014345 |
| *polr1d* | 0.721823 | 0.032707 |
| *rnf2* | -1.12964 | 3.91E-05 |
| *runx3* | 1.718195 | 3.94E-18 |
| *s1pr2* | 0.415986 | 0.024007 |
| *scfd1* | 0.299537 | 0.028434 |
| *sec23a* | -1.43718 | 3.77E-11 |
| *sec24d* | 0.910752 | 0.005705 |
| *sharpin* | -1.43559 | 2.67E-06 |
| *shh* | 1.07417 | 0.004793 |
| *smo* | -0.46599 | 0.039733 |
| *sphk2* | 0.967353 | 7.29E-07 |
| *tfap2a* | 0.81934 | 0.008711 |
| *tshz2* | -0.68911 | 0.000809 |
| *wls* | -1.49603 | 4.15E-07 |
| *wnt4* | 0.983625 | 0.020119 |
| *xylt1* | 0.661423 | 0.009892 |
