# Supplementary material for: Hybrid gene misregulation in multiple developing tissues within a recent adaptive radiation of Cyprinodon pupfishes
Source: PLoS One. 2019 Jul 10;14(7):e0218899. doi: 10.1371/journal.pone.0218899 (PMC6619667; doi:10.1371/journal.pone.0218899)
Supplement: S6 Table — (DOCX) [file pone.0218899.s006.docx]

**Table S6.** Gene ontologies enriched for 6,590 genes misregulated between hybrids and parental species in craniofacial tissue collected at 17-20 dpf (*P* < 0.05; geneontology.org).

| GO:0002181 | GO:0006417 | GO:0044257 | GO:0150063 | GO:0071840 |
| --- | --- | --- | --- | --- |
| GO:0042255 | GO:0006364 | GO:0033554 | GO:0009056 | GO:0051246 |
| GO:0042273 | GO:0043603 | GO:0034613 | GO:1901575 | GO:0006950 |
| GO:0006402 | GO:0034248 | GO:0070727 | GO:0048562 | GO:0043412 |
| GO:0051236 | GO:0033365 | GO:0016567 | GO:0010605 | GO:0036211 |
| GO:0006412 | GO:0006396 | GO:0030163 | GO:1901137 | GO:0006464 |
| GO:0000956 | GO:0048701 | GO:0046700 | GO:0034622 | GO:0022607 |
| GO:0050658 | GO:0010467 | GO:0008104 | GO:0009790 | GO:0016043 |
| GO:0050657 | GO:1904888 | GO:0046907 | GO:0034654 | GO:0051173 |
| GO:0043043 | GO:0034470 | GO:0090304 | GO:0044267 | GO:0010604 |
| GO:0015931 | GO:0016072 | GO:1901361 | GO:0048598 | GO:0009893 |
| GO:0006401 | GO:0048704 | GO:0071705 | GO:0044260 | GO:0009888 |
| GO:0034976 | GO:0010498 | GO:0044270 | GO:0048568 | GO:0048856 |
| GO:0006403 | GO:0070647 | GO:0034641 | GO:0048880 | GO:0007275 |
| GO:0006366 | GO:0034660 | GO:0006325 | GO:0019438 | GO:0048731 |
| GO:0006605 | GO:0043161 | GO:0044249 | GO:0018130 | GO:0032502 |
| GO:0007034 | GO:0034655 | GO:0051649 | GO:0006082 | GO:0019222 |
| GO:0022618 | GO:0044265 | GO:1901576 | GO:0044237 | GO:0009653 |
| GO:0072594 | GO:0009059 | GO:0001501 | GO:0006807 | GO:0010468 |
| GO:0043604 | GO:0034645 | GO:0009058 | GO:0009887 | GO:0060255 |
| GO:0090150 | GO:0006886 | GO:0006139 | GO:0043170 | GO:0031323 |
| GO:0006518 | GO:0006520 | GO:0043009 | GO:0031324 | GO:0006810 |
| GO:0071826 | GO:0010608 | GO:0006725 | GO:0045935 | GO:0051234 |
| GO:0016197 | GO:0043632 | GO:0009792 | GO:1901135 | GO:0051171 |
| GO:0016579 | GO:0019941 | GO:0046483 | GO:0007423 | GO:0080090 |
| GO:0051169 | GO:0048193 | GO:0033036 | GO:0006508 | GO:0051179 |
| GO:0006913 | GO:1901566 | GO:0018193 | GO:0044238 | GO:0009987 |
| GO:0016570 | GO:0015031 | GO:0051641 | GO:0044281 | GO:0032501 |
| GO:0042254 | GO:0048705 | GO:1901360 | GO:0008152 | GO:0008150 |
| GO:0022613 | GO:0048706 | GO:0051276 | GO:1901362 | GO:0050896 |
| GO:0016569 | GO:0015833 | GO:0006259 | GO:0051172 | GO:0007165 |
| GO:0000398 | GO:0044271 | GO:0002520 | GO:0071704 | GO:0007154 |
| GO:0000377 | GO:0006511 | GO:0044248 | GO:0019538 | GO:0023052 |
| GO:0000375 | GO:0045184 | GO:0010629 | GO:1901564 | GO:0050877 |
| GO:0070646 | GO:0032446 | GO:0048534 | GO:0016192 | GO:0006955 |
| GO:0061919 | GO:0009057 | GO:0030097 | GO:0044085 | GO:0046777 |
| GO:0006914 | GO:0042886 | GO:0019752 | GO:0065003 | GO:0099537 |
| GO:0008380 | GO:0016070 | GO:0071702 | GO:0006996 | GO:0099536 |
| GO:0017038 | GO:0006974 | GO:1901565 | GO:0055114 | GO:0007268 |
| GO:0016071 | GO:0006281 | GO:0009892 | GO:0032268 | GO:0098916 |
| GO:0006397 | GO:0019439 | GO:0043436 | GO:0043933 | GO:0007187 |
| GO:0006457 | GO:0051603 | GO:0001654 | GO:0048513 | GO:0007186 |
