# Supplementary figures and images for: Hybrid gene misregulation in multiple developing tissues within a recent adaptive radiation of Cyprinodon pupfishes
Source: PLoS One. 2019 Jul 10;14(7):e0218899. doi: 10.1371/journal.pone.0218899 (PMC6619667; doi:10.1371/journal.pone.0218899)

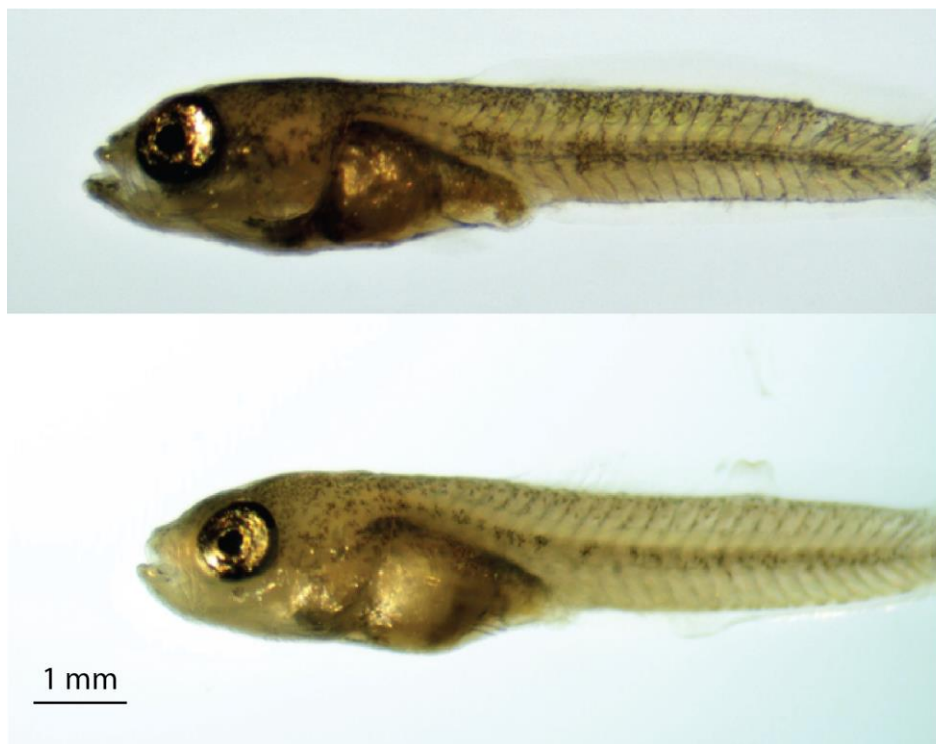

**Fig S1.** 20 day old generalist (top) and molluscivore (bottom).

Supplement: S1 Fig — 20 day old generalist (top) and molluscivore (bottom). (PDF) [file pone.0218899.s007.pdf]
