# Supplementary material for: Hybrid gene misregulation in multiple developing tissues within a recent adaptive radiation of Cyprinodon pupfishes
Source: PLoS One. 2019 Jul 10;14(7):e0218899. doi: 10.1371/journal.pone.0218899 (PMC6619667; doi:10.1371/journal.pone.0218899)
Supplement: S2 Fig — Proportion of reads assigned to features (yellow), unassigned due to multi-mapping (red), and unassigned due to no match to annotated features (blue) using STAR aligner. (PDF) [file pone.0218899.s008.pdf]

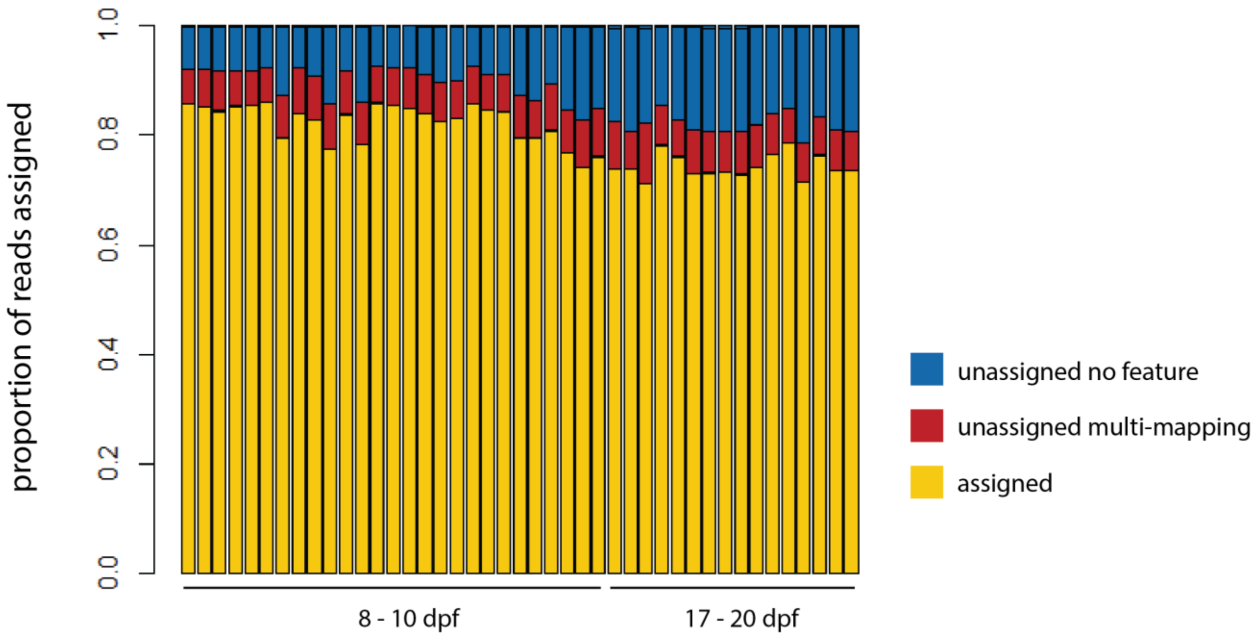

Figure S2. Proportion of reads assigned to features (yellow), unassigned due to multi-mapping (red), and unassigned due to no match to annotated features (blue) using STAR aligner.
