# Supplementary material for: Hybrid gene misregulation in multiple developing tissues within a recent adaptive radiation of Cyprinodon pupfishes
Source: PLoS One. 2019 Jul 10;14(7):e0218899. doi: 10.1371/journal.pone.0218899 (PMC6619667; doi:10.1371/journal.pone.0218899)
Supplement: S3 Fig — The first and second principal component axes accounting for a combined 75% of the total variation between generalist (red), molluscivore (blue), and hybrid (purple) samples across reads mapped to annotated features. Point shape indicates the sequencing date of the sample. (PDF) [file pone.0218899.s009.pdf]

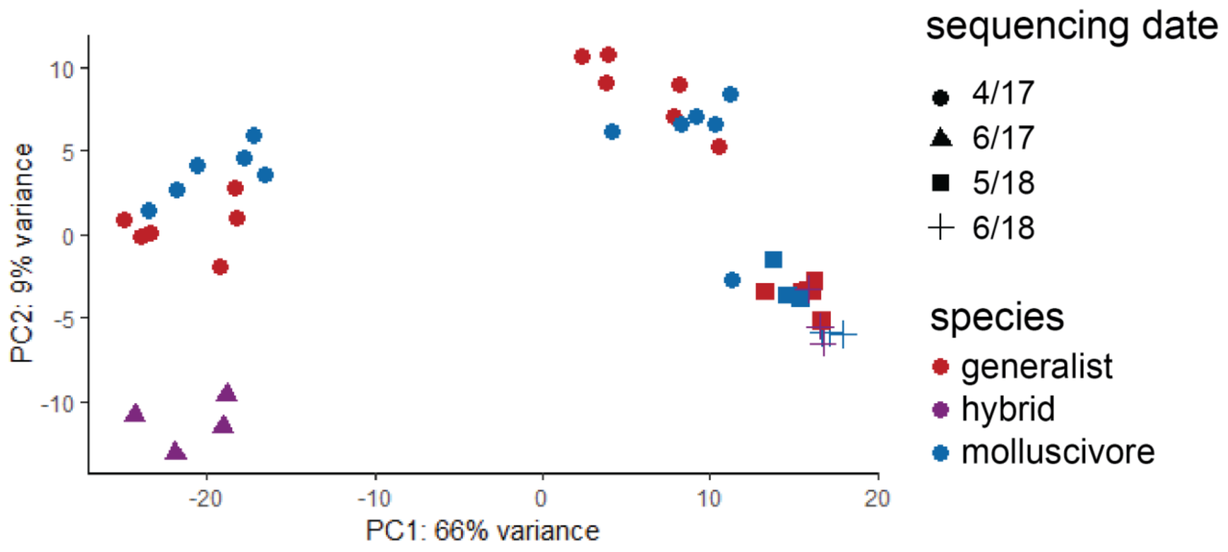

Figure S3. The first and second principal component axes accounting for a combined 75% of the total variation between generalist (red), molluscivore (blue), and hybrid (purple) samples across reads mapped to annotated features. Point shape indicates the sequencing date of the sample.
