# Supplementary material for: Hybrid gene misregulation in multiple developing tissues within a recent adaptive radiation of Cyprinodon pupfishes
Source: PLoS One. 2019 Jul 10;14(7):e0218899. doi: 10.1371/journal.pone.0218899 (PMC6619667; doi:10.1371/journal.pone.0218899)
Supplement: S4 Fig — Genes showing underdominant expression in hybrids show a higher magnitude of misregulation than genes showing overdominance (Wilcoxon rank sum test; 8 dpf P = 8.5 × 10^−5^ 17–20 dpf P < 2.2 × 10^−16^). (PDF) [file pone.0218899.s010.pdf]

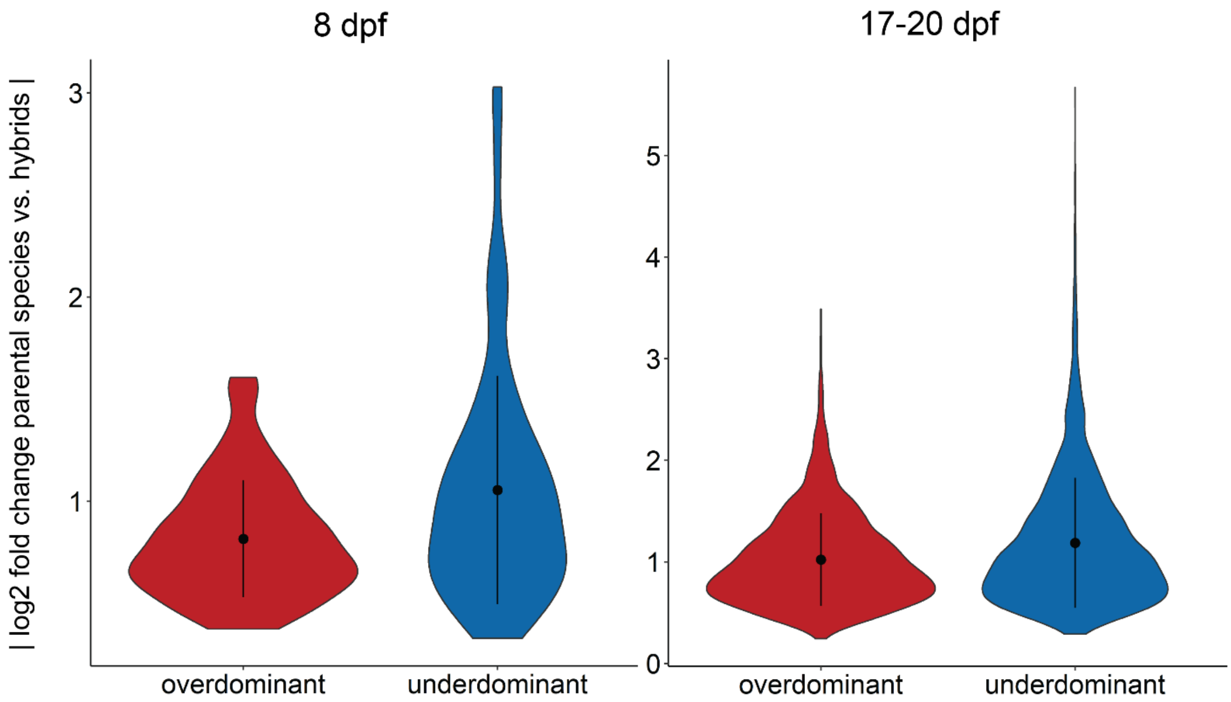

Fig S4. Genes showing underdominant expression in hybrids show a higher magnitude of misregulation than genes showing overdominance (Wilcoxon rank sum test; 8 dpf $P = 8.5 \times 10^{-5}$ 17-20 dpf $P < 2.2 \times 10^{-16}$).
