# Supplementary material for: Hybrid gene misregulation in multiple developing tissues within a recent adaptive radiation of Cyprinodon pupfishes
Source: PLoS One. 2019 Jul 10;14(7):e0218899. doi: 10.1371/journal.pone.0218899 (PMC6619667; doi:10.1371/journal.pone.0218899)
Supplement: S5 Fig — The 8 dpf hybrids were sequenced at the same facility with the same library kit as the 17–20 dpf hybrids, while the 8–10 dpf parental species were sequenced at the same facility with the same library kit as the 17–20 dpf parental species. (A) The comparison between 8 dpf parental species and 8 dpf hybrids revealed 370 genes (2.1%) misregulated. (B) The comparison between 8 dpf hybrids and 8–10 dpf parental species revealed 997 (6%) genes misregulated–a 37% increase. We used this inflated estimate to adjust our estimate of misregulation in 17–20 dpf hybrid craniofacial tissues. Red points indicate genes detected as differentially expressed at 5% false discovery rate with Benjamini-Hochberg multiple testing adjustment. Grey points indicate genes showing no significant difference in expression between groups. (PDF) [file pone.0218899.s011.pdf]

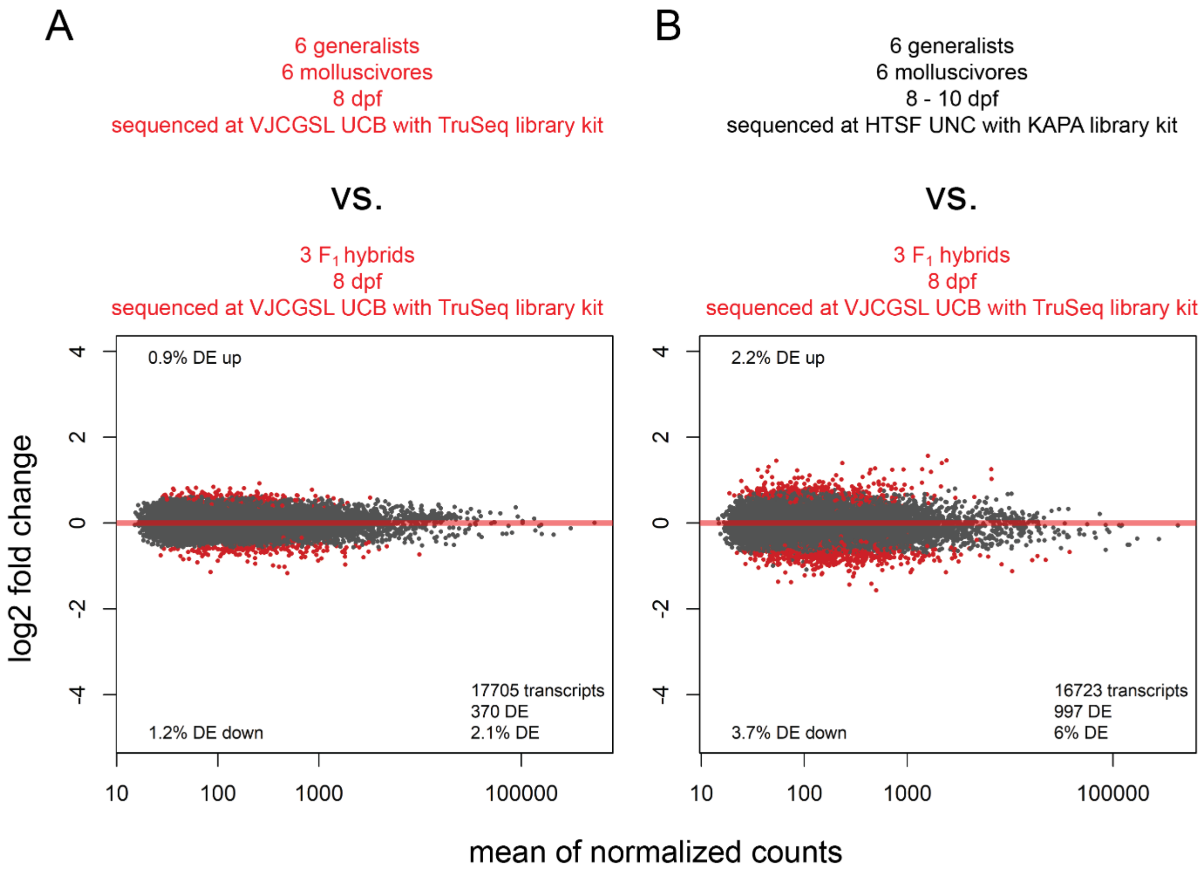

Fig S5. Estimating the effect of sequencing design on the proportion of genes misregulated in hybrids. The 8 dpf hybrids were sequenced at the same facility with the same library kit as the 17-20 dpf hybrids, while the 8-10 dpf parental species were sequenced at the same facility with the same library kit as the 17-20 dpf parental species. A) The comparison between 8 dpf parental species and 8 dpf hybrids revealed 370 genes (2.1%) misregulated. B) The comparison between 8 dpf hybrids and 8-10 dpf parental species revealed 997 (6%) genes misregulated – a 37% increase. We used this inflated estimate to adjust our estimate of misregulation in 17-20 dpf hybrid craniofacial tissues. Red points indicate genes detected as differentially expressed at 5% false discovery rate with Benjamini-Hochberg multiple testing adjustment. Grey points indicate genes showing no significant difference in expression between groups.
