# Supplementary material for: Hybrid gene misregulation in multiple developing tissues within a recent adaptive radiation of Cyprinodon pupfishes
Source: PLoS One. 2019 Jul 10;14(7):e0218899. doi: 10.1371/journal.pone.0218899 (PMC6619667; doi:10.1371/journal.pone.0218899)
Supplement: S6 Fig — We did not find significant differences between 17–20 dpf hybrid craniofacial samples and samples sequenced on other dates for (A) median percent GC content across reads, (B) number of normalized read counts, or (C) number of raw fastq reads. the proportion of duplicate reads for each sample (Pairwise Welch two sample t test; P < 0.0001 = ****, *** = 0.001, ** = 0.01, * = 0.05). (PDF) [file pone.0218899.s012.pdf]

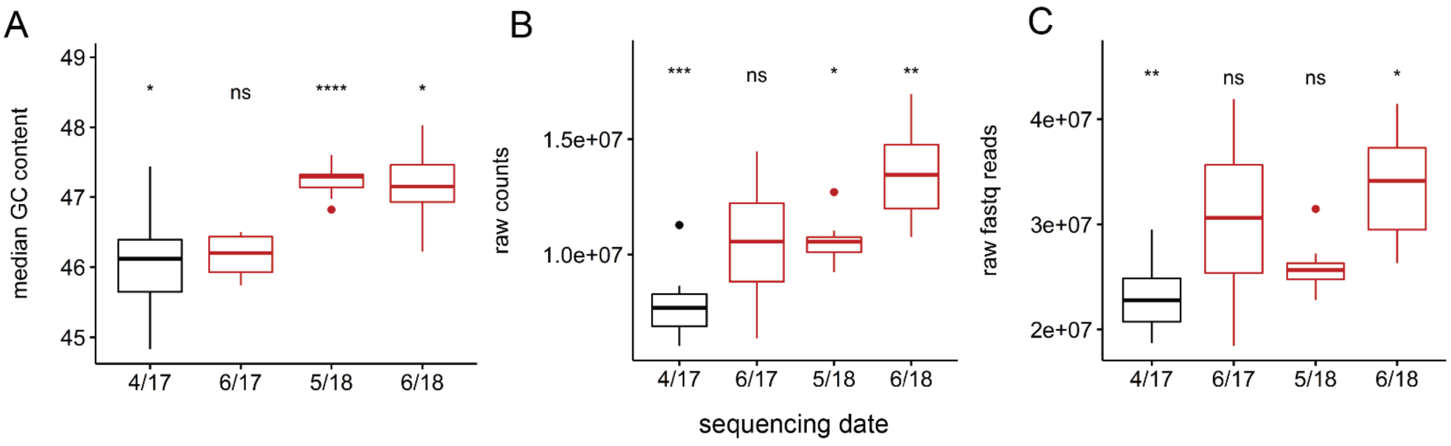

Fig S6. We did not find significant differences between 17-20 dpf hybrid craniofacial samples and samples sequenced on other dates for A) median percent GC content across reads, B) number of normalized read counts, or C) number of raw fastq reads. the proportion of duplicate reads for each sample (Pairwise Welch two sample t test; $P < 0.0001$ = ****, *** = 0.001, ** = 0.01, * = 0.05).
