# Supplementary material for: Hybrid gene misregulation in multiple developing tissues within a recent adaptive radiation of Cyprinodon pupfishes
Source: PLoS One. 2019 Jul 10;14(7):e0218899. doi: 10.1371/journal.pone.0218899 (PMC6619667; doi:10.1371/journal.pone.0218899)
Supplement: S1 Appendix — Examples of command line arguments and DESeq2 scripts for bioinformatics pipelines. (DOCX) [file pone.0218899.s013.docx]

**Coding Supplement**

# trim adaptors

trim_galore -q 20 --paired --illumina file1.fastq file2.fastq

# align reads to features

star --runThreadN 4 --genomeDir /genome_dir --readFilesIn file1.fastq file2.fastq --outFileNamePrefix file"

# count reads across features

featureCounts -p -a file.gtf -B -C -G file.fasta -s 2 -T 4 -o files.bam

# haplotype caller

java -Xmx10g -jar GenomeAnalysisTK.jar -T HaplotypeCaller -drf DuplicateRead -R file.fasta -I file.bam -dontUseSoftClippedBases -stand_call_conf 20.0 -nct 8 -o file.g.vcf

# merge vcfs

vcf-merge file1.g.vcf.gz file2.g.vcf.gz > merged_raw_variants.vcf

# select snps

java -jar GenomeAnalysisTK.jar -T SelectVariants -R file.fasta -V raw_variants.vcf -selectType SNP -o raw_snps.vcf

# filter snps

java -jar GenomeAnalysisTK.jar -T VariantFiltration -R file.fasta -V raw_snps.vcf --filterExpression "QD < 2.0 || FS > 60.0 || MQ < 40.0 || MQRankSum < -12.5 || ReadPosRankSum < -8.0" --filterName "standard_snp_filter" -o filtered_snps.vcf

# filter minor allele frequency 5% and max missing genotype 90%

vcftools --vcf filtered_snps.vcf --maf 0.05 --max-missing 0.9 --recode --out maf_0.5_maxmiss_0.9_snps"

# phase

java -jar GenomeAnalysisTK.jar -T ReadBackedPhasing -R file.fasta -I file.bam --variant snps.vcf -o phased.vcf --phaseQualityThresh 20.0

# count allele reads

java -jar GenomeAnalysisTK.jar -R file.fasta -T ASEReadCounter -U ALLOW_SEQ_DICT_INCOMPATIBILITY -o allele_counts.csv -I file.bam -sites phased.vcf

# output snp genotypes

java -jar GenomeAnalysisTK.jar -R file.fasta -T VariantsToTable -V phased.vcf -F CHROM -F POS -GF GT -GF HP -o snp_table.txt

# Basic DESeq2 design

dds <- DESeqDataSetFromMatrix(countData = cts,

colData = colData,

design= ~species)

dds <- estimateSizeFactors(dds)

idx <- rowSums(counts(dds, normalized=TRUE) >= 10 ) >= (nrow(colData))

dds <- dds[idx,]

dds <- DESeq(dds)
